# Elevated plasma miR-133b and miR-221-3p as biomarkers for early Parkinson’s disease

**DOI:** 10.1038/s41598-021-94734-z

**Published:** 2021-07-27

**Authors:** Qihua Chen, Na Deng, Ke Lu, Qiao Liao, Xiaoyan Long, Deming Gou, Fangfang Bi, Jinxia Zhou

**Affiliations:** 1 Department of Neurology, Xiangya Hospital, Central South University, Changsha, 41000 China; 2 Shenzhen Key Laboratory of Microbial Genetic Engineering, College of Life Sciences and Oceanography, Guangdong Provincial Key Laboratory of Regional Immunity and Diseases, Carson International Cancer Center, Shenzhen University, Shenzhen, 518060 Guangdong China

**Keywords:** Parkinson's disease, High-throughput screening, Biomarkers

## Abstract

Blood circulating microRNAs (miRNAs) are proposed to be promising biomarkers for many neurodegenerative disorders, including Parkinson’s disease (PD). However, there is a lack of identified differentially expressed miRNAs in PD from different studies. The aim of this study was to evaluate miRNAs expression in PD. We measured plasma circulating miRNA expression in three independent sets with a total of 151 PD patients, 21 multiple system atrophy (MSA) patients and 138 healthy controls using high-throughput RT-PCR. We identified that elevated miR-133b and miR-221-3p discriminated early-stage PD from controls with 94.4% sensitivity and 91.1% specificity. Elevated miR-133b and miR-221-3p distinguished PD from controls with 84.8% sensitivity and 88.9% specificity. In addition, miR-4454 distinguished PD from MSA with 57.1% sensitivity and 82.6% specificity. Hence, elevated miR-133b and miR-221-3p potentially represent good biomarkers for early PD, and a combination of miR-133b, miR-221-3p and miR-4454 has the potential to serve as a non-invasive biomarker for PD diagnosis.

## Introduction

The diagnosis of Parkinson’s disease (PD) is difficult, particularly in early stages. Approximately one-third of the clinical diagnoses in patients with parkinsonism are revised within, on average, the first five years of disease onset^1^. Additionally, other than limited clinical and neuroimaging evaluations, which are typically expensive, there is no established objective and efficient method to follow PD progression or evaluate the efficacy of treatments. Hence, seeking novel and reliable molecular biomarkers of PD is meaningful.

MicroRNAs (miRNAs) are a class of non-coding RNAs post-transcriptionally regulate the expression of target genes. MiRNAs circulate in a stable and cell-free form in serum, plasma and cerebral spinal fluid (CSF) and are involved in cell differentiation, proliferation, and apoptosis in many diseases, including neurodegenerative diseases^2^. The miRNA expression profile differs between healthy and diseased tissue, and blood circulating miRNA quantification as a biomarker has been explored in several diseases, including certain types of cancers^3^ and cardiovascular disease^4^. However, miRNAs as biomarkers for neurodegenerative disorders, including PD, remains uncertain. A limited number of studies have been published on miRNA expression in biological fluids from PD patients, and only a small proportion of human miRNAs have been detected. Furthermore, the reported differentially expressed miRNAs barely overlapped among different studies^5,6^. A portion of these discrepancies were attributed to the methodologies of sample preparation and measurement. Although PD is a central nervous system disorder, the peripheral component of PD has attracted considerable attention^7^. Non-motor symptoms of salivation problems and constipation seem to correlate with changes in peripheral innervation and α-synuclein pathology^8^. Braak hypothesizes that α-synuclein pathology starts in the periphery and subsequently spreads to the upper brain through peripheral nerves^9^. Hence, peripheral blood and its derivatives are proposed as good sources for PD biomarker exploration. Plasma and serum are the two most common components of blood for biomarker studies. Previous studies have indicated that plasma miRNAs are closer to the true repertoire of circulating miRNAs^10^. However, the majority of the archived samples for miRNA biomarker research are stored as serum since plasma miRNAs are present in lower concentrations, thereby necessitating higher requirements for detection methods.

Quantitative real-time PCR (qRT-PCR) is one of the most sensitive techniques for miRNA detection. The most popular stem-loop method of qRT-PCR requires unique reverse transcription primers and a specific probe for each miRNA assay, which has some limitations for high-throughput screening. Our group has previously developed a novel S-Poly(T)Plus for miRNA qPCR assays, which combines polyadenylation and reverse transcription in one step while retaining the S-Poly(T)primer^11^. The S-Poly(T) Plus method has been shown to be a simple and sensitive miRNA profiling tool for detecting circulating miRNAs with precise quantification^11,12^.

In the current study, we presented a proof-of-concept study to demonstrate the feasibility of utilizing plasma circulating miRNAs for PD biomarker discoveries. First, the human plasma miRNA profile was analyzed in 78 PD and 78 normal controls using S-Poly(T) Plus qRT-PCR, and the seven most differentially expressed miRNAs were selected. The seven miRNAs were further assessed by qRT-PCR in a new duplication set of 27 PD patients and 15 controls, and three differentially expressed miRNAs (miR-133b, miR-221-3p, miR-320a) were identified. Finally, seven candidate miRNAs were verified in a new cohort of 46 PD patients, 21 multiple system atrophy (MSA) patients and 45 healthy controls. Elevated miR-133b and miR-221-3p differentiated early-stage PD from controls; moreover, the combination of miR-133b, miR-221-3pand miR-4454 discriminated PD from healthy controls and MSA patients.

## Results

### Screening of candidate miRNAs in PD

**Figure 1:**
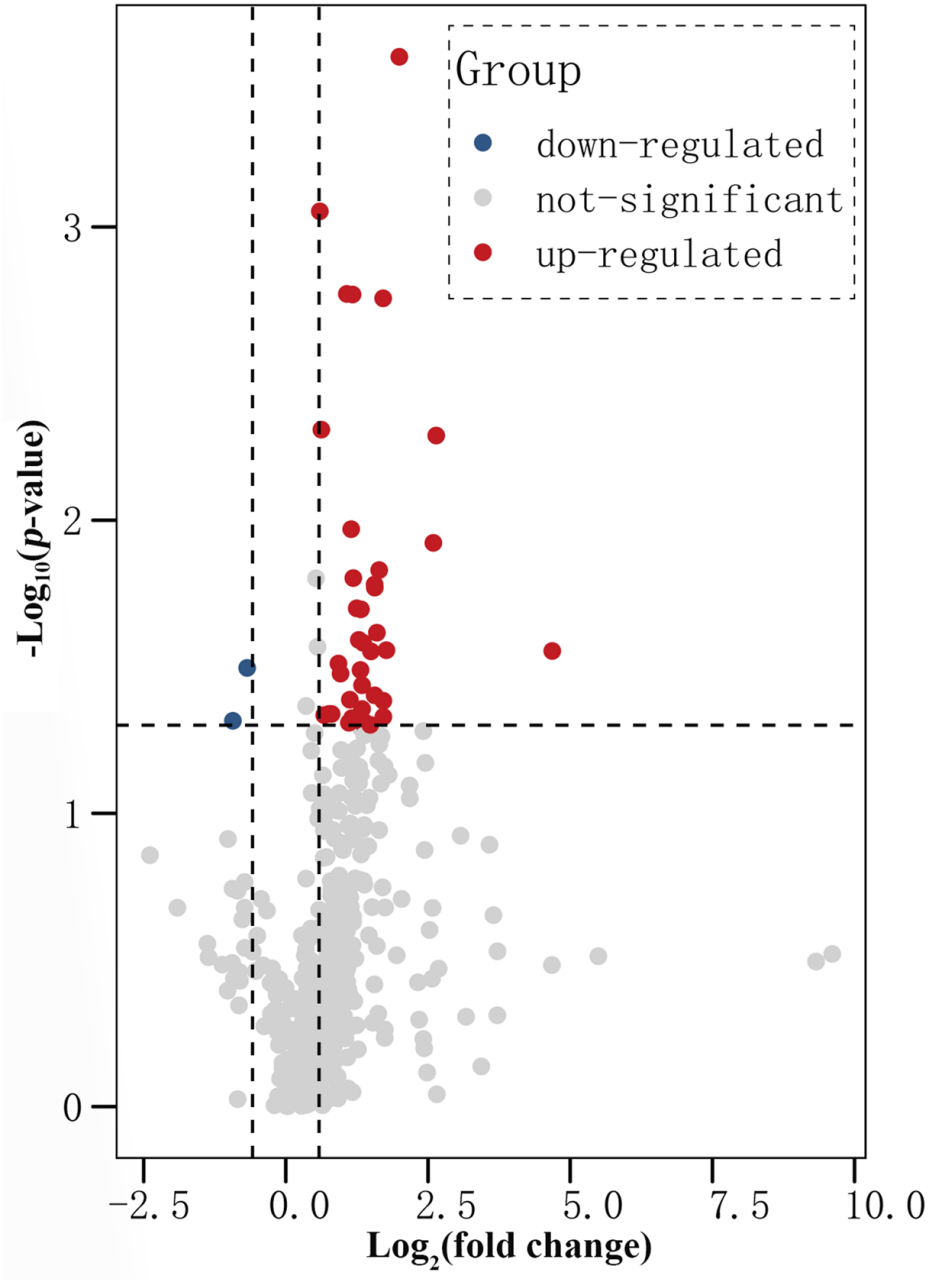
Volcano plot illustrating 486 miRNAs differentially expressed between Parkinson’s disease cases (n = 78) and healthy controls (n = 78) from cohort 1. A paired t-test was used to identify significantly differentially expressed miRNAs.

Based on the S-Poly(T) Plus miRNA assay, qRT-PCR was utilized to detect human plasma miRNA profiles (486 miRNAs) between 78 PD patients and 78 controls with three pools (Fig. 1). The demographic characteristics of the cases are shown in Table 1. In this study, exogenous cel-miR-54-5p was added during the RNA extraction process as a normalization control. Then, highly and consistently expressed miRNAs were selected as candidate endogenous reference genes. The stability of the candidate reference genes was further evaluated by two different algorithms (geNorm and NormFinder). Hsa-miR-25-3p was finally found to be quantifiable and stable in all samples, demonstrating no significant differential expression between groups (*p* > 0.05) and less variation between inter- and intra-group. Hence, hsa-miR-25-3p was used as an endogenous reference gene in this study. All the following assessments were double-checked with cel-miR-54-5p and Hsa-miR-25-3p as exogenous and endogenous reference miRNAs, respectively, and the results from these two reference miRNAs were independently consistent. Hence, all data presented in this study were normalized to cel-miR-54-5p.

**Table 1:** Demographic and clinical characteristics.

Cohort 1		Controls		PD		
Total N		78		78		
Males		40		42		
Females		38		36		
Early-stage PD		–		44		
Advanced-stage PD		–		34		
Age (years)		59.68 (58.22-60.14)		60.80 (58.64–62.96)		
Duration (years)				5.14 (4.03–6.25)		
Modified H-Y stage		–		2.69 (2.48–2.9)		
UPDRS-total		–		23.88 (21.22–26.54)		
UPDRS-III		–		41.18 (36.66–45.7)		

Cohort 2	Controls		PD			
			Total		Treated	Untreated
Total N	15		27		14	13
Males	7		13		7	7
Females	8		14		7	6
Early-stage PD	–		18		8	10
Advanced-stage PD	–		9		5	4
Age (years)	59.92 (55.32–64.52)		60.11 (56.51–63.71)		59.71 (54.39–65.03)	60.54 (55.46–65.62)
Duration(years)			2.63 (2.11–3.15)		2.29 (1.66–2.92)	3 (2.17–3.83)
Modified H–Y stage			2.37 (2.02–2.72)		2.29 (1.79–2.79)	2.46 (1.96–2.96)
UPDRS–total			20.04 (16.46–23.54)		19.71 (15.29–24.13)	20.38 (14.7–26.06)
UPDRS-III			34.93 (28.23–41.63)		35.43 (27.45–43.41)	34.38 (23.1–45.66)
NMSQ scores			8.15 (6.53–9.77)		7.71 (5.01–13.41)	8.62 (6.82–10.42)

Cohort 3	Controls		PD		MSA	
Total N	45		46		21	
Males	18		13		7	
Females	27		33		14	
Early-stage PD	–		18		–	
Advanced-stage PD	–		28		–	
Age (years)	61.54 (59.96–63.12)		63.09 (60.19–65.99)		61.86 (58.75–64.97)	
Duration (years)			5.72 (4.43–7.01)		3.05 (1.73–4.37)	
Modified H-Y stage	–		2.86 (2.57–3.15)		–	
UPDRS-total	–		28.89 (25.18–32.60)		–	
UPDRS-III	–		55.46 (48.75–62.17)		–	

PD, Parkinson’s disease; H-Y stage, Hoehn and Yahr stage; UPDRS, Unified Parkinson's Disease Rating Scale; UPDRS-III, Unified Parkinson's Disease Rating Scale Part III; NMSQ,* nonmotor system questionnaire*; early-stage PD,* modified* H-Y stage 1–2.5; advanced-stage PD, modified H-Y stage 3-5.

Analysis of miRNAs profile showed 32 miRNAs significantly dysregulated in PD compared with controls, including two down-regulated (fold changes ≤ 0.65, *p* ≤ 0.05) and 30 up-regulated (fold changes ≥ 1.5, *p* ≤ 0.05). The results were summarized in Table 2. Among these 32 miRNAs, miR133b^13,14^, miR-320^15,16^, miR-221-3p^14^, miR-627-5p^17^ and miR-205^18^ had been proposed to involve in the pathogenesis of PD from literature review or bioinformatic analysis. In addition, they were also shown in relative low Ct value (≤ 33) ,large fold changes (> 2 or < 0.65, Table 2), and *p *value < 0.05 in screening test, indicating as good candidate miRNAs. Considering four miR-320 families (miR-320a/b/c/d) were recruited from the screening set, the miR-320a, which was proposed to have the strongest relationship with PD^15,16^ and presented with the largest change fold and lowest Ct value in this family (change fold = 3.03, Ct value = 23, Table 2), was selected as a symbol for miR-320 family. Furthermore, miR-432-5p and miR-4454 were also selected as candidates as they had the largest change folds or lowest Ct value among these 32 miRNAs with good *p *value (Table 2). Hence, seven miRNAs (miR-432-5p, miR-133b, miR-320a, miR-4454, miR-221-3p, miR-627-5p and miR-205) were selected as candidate miRNAs for further validation.

**Table 2:** Differentially expressed miRNAs in Parkinson’s disease compared to controls.

miRNAs	Ct value	Fold change	*p *Value
miR-432-5p*	31	3.4	0.028
miR-23a-5p	32	3.39	0.047
miR-133b*	28	3.28	0.041
miR-3177-3p	35	3.27	0.0018
miR-320a*	23	3.03	0.024
miR-320b	25	2.95	0.017
miR-625-5p	31	2.95	0.017
miR-491-5p	32	2.95	0.04
miR-4454*	23	2.82	0.028
miR-221-3p*	24	2.8	0.05
miR-30d-3p	34	2.62	0.049
miR-320c	24	2.57	0.026
miR-324-5p	29	2.56	0.049
miR-625-3p	32	2.53	0.037
miR-339-3p	31	2.53	0.044
miR-130b-3p	29	2.5	0.02
miR-320d	24	2.49	0.032
miR-548a-3p	34	2.43	0.026
miR-627-5p*	33	2.27	0.016
miR-1260b	26	2.26	0.0017
miR-185-5p	24	2.24	0.047
miR-503-5p	32	2.22	0.011
miR-210-3p	30	2.18	0.041
miR-197-3p	28	2.16	0.049
miR-4286	26	2.1	0.002
miR-4306	30	1.9	0.031
miR-92b-3p	31	1.75	0.046
miR-3621	26	1.69	0.046
miR-3972	34	1.6	0.046
miR-1538	34	1.51	0.0009
miR-205*	34	0.62	0.032
miR-10b-5p	31	0.53	0.048

*Selected candidate miRNAs.

### Duplication of miRNAs biomarkers in PD

Twenty-seven PD cases (including 13 L-dopa-naïve PD and 14 L-dopa-treated PD) and 15 age- and sex-matched controls were recruited in this set. There were no statistically significant differences in age, sex, disease duration, modified Hoehn and Yahn (H-Y) stage or UPDRS scores were noted between the L-dopa-naïve and L-dopa-treated PD groups. The demographic characteristics of the cases are shown in Table 1*.*

#### L-dopa naïve PD versus L-dopa treated PD

As previously reported, L-dopa and other drug treatments may alter miRNA expression in PD^19^. Hence, we first aimed to determine whether PD drug treatments would affect the expression of seven candidate miRNAs. Statistically significant differences in the seven candidate miRNA levels were not noted between the L-dopa-naïve and L-dopa-treated PD groups (*t *test, *p* > 0.05, Supplementary Fig. 1), indicating that L-dopa treatments probably have minimal effect on the expression of these candidate miRNAs.

#### PD versus controls

**Figure 2:**
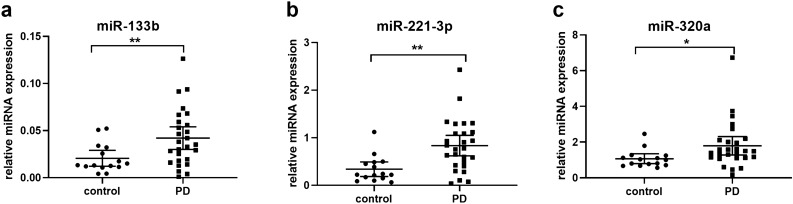
Plasma miR-133b (**a**), miR-221-3p (**b**) and miR-320a (**c**) expression in patients with PD (n = 27) and healthy controls (n = 15) from cohort 2. An independent *t* test was used to evaluate differences between groups. Data are presented as the means ± SEMs. The results are indicated as **p value < 0.01 and *p value < 0.05. PD, Parkinson’s disease.

Furthermore, the levels of seven candidate miRNAs were assessed between 27 PD cases and 15 controls. The levels of miR-133b, miR-221-3p and miR-320a were significantly higher in PD cases compared to controls (for miR-133b, 2.04 times increase, *t *test, *p* = 0.004; for miR-221-3p, 2.47 times increase, *t *test, *p* < 0.001; for miR-320a, 1.68 times increase, *t *test, *p* = 0.014; Fig. 2). There was no significant difference in the expression of miR-205, miR-432-5p, miR-4454 and miR-627-5p between the PD group and controls (*t* test, *p* > 0.05). No age- or sex-dependent difference in the expression of the seven candidate miRNAs was found between the control or PD groups. Analyses of covariance between groups with sex and age as covariates showed sex and age did not exert significant effects on the considered miRNAs.

**Figure 3:**
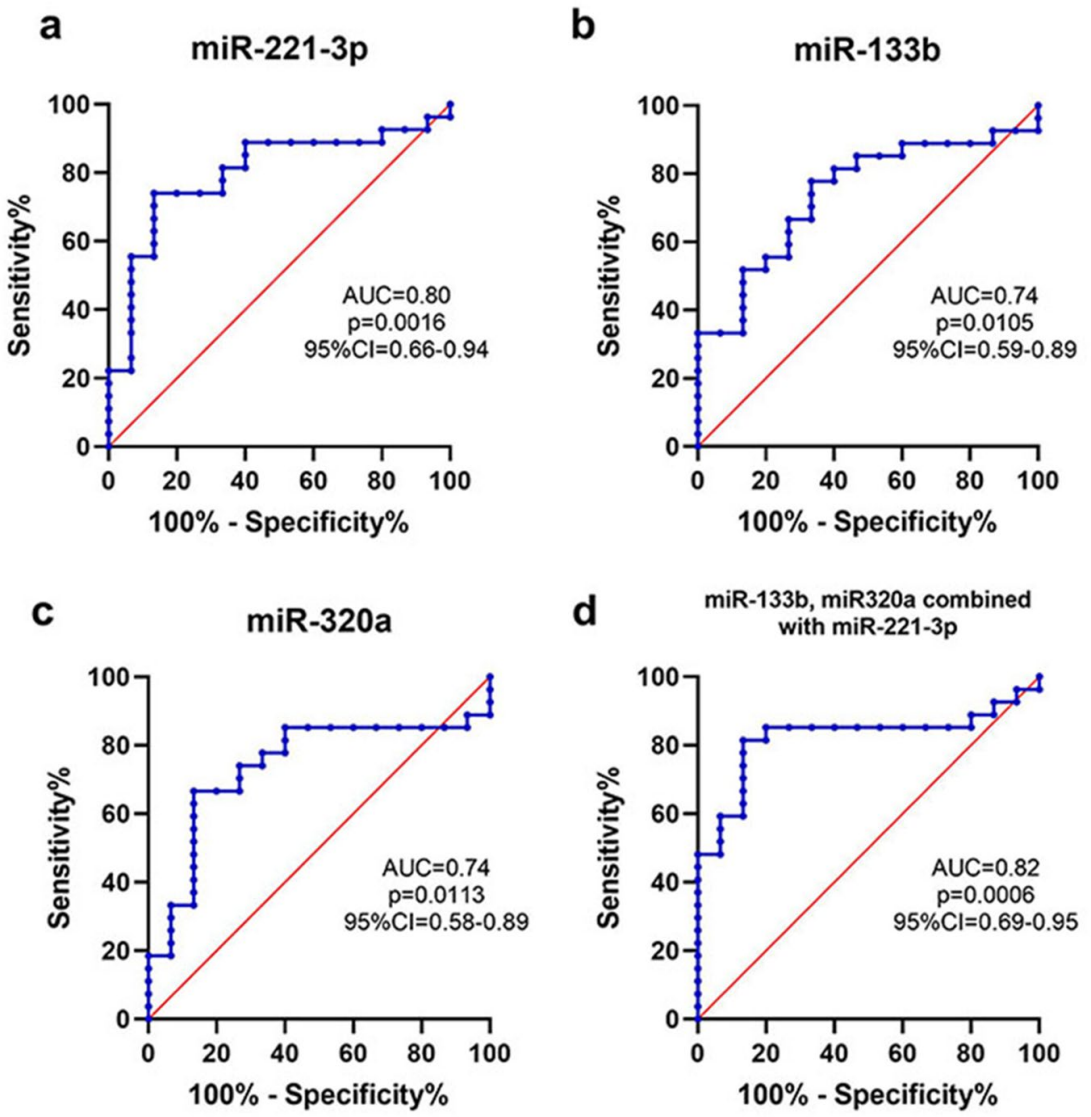
ROC analysis of plasma miRNAs as PD biomarkers. (**a**-**d**) ROC curves of miR-221-3p (**a**), miR-133b (**b**), miR-320a (**c**) and combination of these three miRNAs (**d**) for discriminating patients with PD (n = 27) from healthy controls (n = 15) in cohort 2. PD, Parkinson’s disease.

The diagnostic accuracy of miRNAs differentially expressed between PD and controls was further assessed by ROC curve analysis. The area under the curve (AUC) value for miR-221-3p was 0.80, with 74.1% sensitivity and 86.7% specificity (Fig. 3a). The AUC value for miR-133b and miR-320a was 0.74 with 77.8% and 66.7% sensitivity and 66.7% and 86.7% specificity, respectively (Fig. 3b,c). Combining these three miRNAs increased the AUC value to 0.82. They sensitivity was 81.5%, and the specificity was 86.7% (Fig. 3d). The diagnostic power of miRNAs to discriminate between PD and healthy controls is summarized in Table 3.

**Table 3:** Diagnostic power of miRNAs to discriminate between PD and healthy controls (measured by ROC curve analyses).

Groups	miRNAs	AUC	95% CI	Cut-off value	Sensitivity (%)	Specificity (%)
PD versus control	miR-320a	0.74	0.58–0.90	1.30	66.7	86.7
	miR-133b	0.74	0.59–0.89	0.018	77.8	66.7
	miR-221-3p	0.80	0.66–0.94	0.50	74.1	86.7
	miR-320a, miR133b and miR-221-3p	0.82	0.69–0.95	0.61	81.5	86.7
Early PD versus control	miR-133b	0.68	0.49–0.86	0.018	66.7	66.7
	miR-221-3p	0.75	0.58–0.93	0.54	72.2	86.7
	miR-133b and miR-221-3p	0.75	0.57–0.93	0.52	77.8	80.0

PD, Parkinson’s disease; AUC, area under the curve; CI, confidence interval.

#### Biomarkers for early PD diagnosis

**Figure 4:**
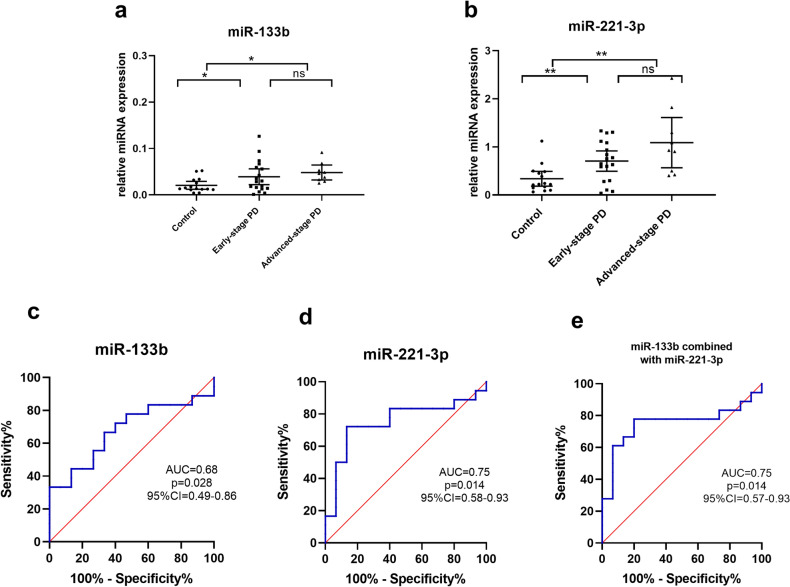
Plasma miR-133b (**a**) and miR-221-3p (**b**) expression in early-stage PD (modified H-Y stage 1–2.5, n = 18), advanced-stage PD (modified H-Y stage 3–5, n = 9) and healthy controls (n = 15) from cohort 2. ROC analysis of plasma miR-133b (**c**), miR-221-3p (**d**) and their combination (**e**) for discriminating patients with early-stage PD from healthy controls in cohort 2. The ANOVA with post-hoc was used to evaluate differences between groups. Data are presented as the means ± SEMs. The results are indicated as **adjust *p* value < 0.01 and *adjust *p* value < 0.05. PD, Parkinson’s disease.

Based on modified H-Y staging, PD cases were divided into early stages (modified H-Y stage 1–2.5) and advanced stages (modified H-Y stage 3–5, Table 1). To find biomarkers for early PD diagnosis, 18 PD cases in early clinical stages were selected for comparison with 15 controls (Table 1). The levels of miR-133b and miR-221-3p were significantly upregulated in early-stage PD compared to controls (for miR-133b, 1.95-fold increase, post-hoc with Bonferroni, adjust *p* = 0.05; for miR-221-3p, 2.09-fold increase, post-hoc with Bonferroni, adjust *p* = 0.007; Fig. 4a,b). No significant differences on the levels of other five candidate mRNA between early-stage PD and controls were found. For early PD diagnosis, the AUC values for miR-133b and miR-221-3p were 0.68 and 0.75, respectively. The sensitivity and specificity of miR-133b were both 66.7%, and those of miR-221-3p were 72.2% and 86.7%, respectively (Fig. 4c,d). The combination of miR-133b and miR-221-3p did not change the AUC value (0.75) and the sensitivity was 77.8%%, and the specificity was 80.0% (Fig. 4e). The diagnostic power of miRNAs to discriminate between early-stage PD and healthy controls is summarized in Table 3.

### Validation of predictive miRNA biomarkers in PD

#### PD versus controls versus MSA

To validate the diagnostic value of seven miRNA candidates, a new independent cohort with 46 PD, 21 MSA and 45 healthy controls was assessed (Table 1) and analyzed.

**Figure 5:**
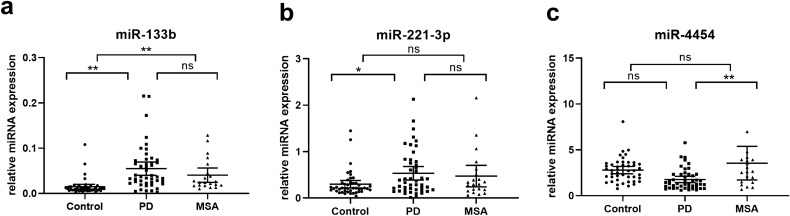
Plasma miR-133b (**a**), miR-221-3p (**b**), and miR-4454 (**c**) expression among patients with PD (n = 46), patients with MSA (n = 21) and healthy controls (n = 45) in cohort 3. The Kruskal–Wallis analysis with post hoc was used to evaluate differences between groups. Data are presented as the means ± SEMs. The results are indicated as ** adjust *p* value < 0.01, *adjust *p* value < 0.05, and ns: no significance. PD, Parkinson’s disease; MSA, multiple system atrophy.

The levels of miRNA-133b, miRNA-221-3p and mi R-4454 were significantly different among control, PD and MSA groups. MiR-133b expression was significantly upregulated in both PD and MSA compared to controls (3.67-fold increase in PD, post-hoc of Kruskal–Wallis analysis, adjust *p* < 0.001; 2.70-fold increase in MSA, post hoc of Kruskal–Wallis analysis, adjust *p* < 0.001; Fig. 5a&c). No significant difference in miRNA-133b expression was noted between PD and MSA (post-hoc of Kruskal–Wallis analysis, adjust *p* = 1.00, Fig. 5a). Meanwhile, miRNA-221-3p levels were significantly upregulated in PD but not in MSA compared to the control (for PD, 1.79-fold increase, post hoc of Kruskal–Wallis analysis, adjust *p* = 0.032; Fig. 5b). Although miR-4454 expression was not significantly different between the PD and control groups, an unexpectedly moderate upregulation of miR-4454 levels was observed in MSA compared to PD (2.00-fold increase, post hoc of Kruskal–Wallis analysis , adjust *p* = 0.008, Fig. 5c). There was no significant difference in miR-205, miR-320a, miR-627-5p and miR-432-5p expression among PD, MSA and controls (Kruskal–Wallis analysis, *p* > 0.05, supplementary Fig. 2). There was no significant difference in miR-320a expression between PD and controls (*p* = 0.058), which was different from the result in duplication set. Higher miRNA-221-3p expression was found in female compared to that in male in PD group (1.81-fold increase in female, Mann–Whitney U test, *p* = 0.025). However, these sex-based differences were not observed in control or MSA group. No sex-dependent difference in the expression of other candidate miRNAs were found in three groups. Analyses of covariance between groups with sex and age as covariates showed sex and age did not exert significant effects on the candidate miRNAs.

**Figure 6:**
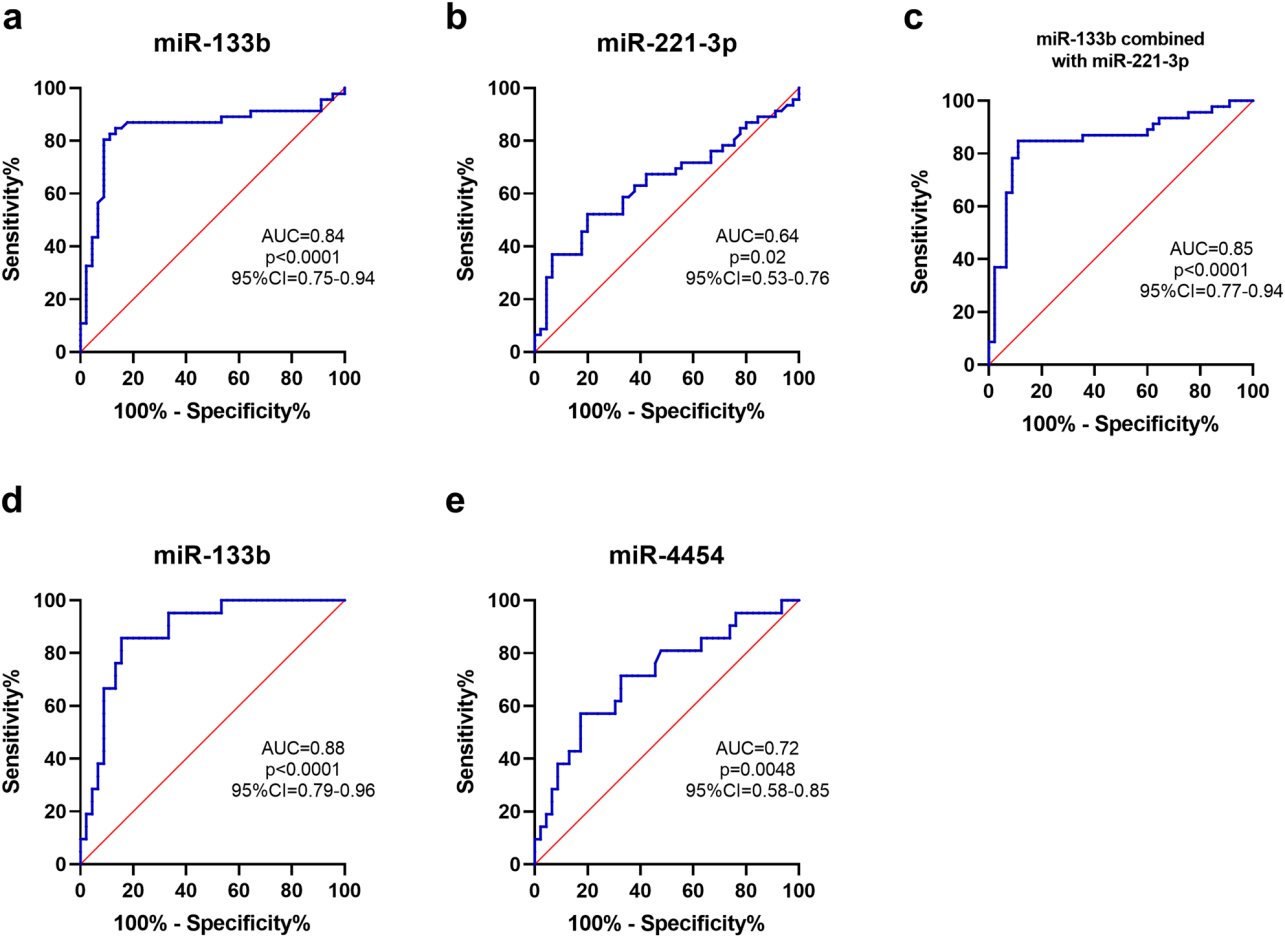
ROC analysis of plasma miRNAs as PD biomarkers. (**a**–**c**) ROC curves of miR-133b (**a**), miR-221-3p (**b**), and their combination (**c**) for discriminating patients with PD (n = 46) from healthy controls (n = 45) in cohort 3. (**d**) ROC curves of miR-133b for discriminating patients with MSA (n = 21) from healthy controls (n = 45) in cohort 3. (e) ROC curves of miR-4454 for discriminating patients with PD (n = 46) from patients with MSA (n = 21) in cohort 3. PD, Parkinson’s disease; MSA, multiple system atrophy.

To investigate the diagnostic value of the candidate miRNAs for PD, logistic regression analysis was performed. Univariate logistic regression found that increased plasma miR-133b (*p* < 0.001), and miR-221-3p (*p* = 0.012*)* differentiated PD patients from healthy controls (Table 4). All factors with a *p *value < 0.05 were subsequently included in the multivariate logistic regression (stepwise) analysis, which demonstrated that miR-133b expression (*p* < 0.001) and miR-221-3p (*p* = 0.041) might also be independent factors for diagnosis of PD (Table 5). ROC curve analysis revealed good diagnostic values for miR-133b to differentiate PD from controls (AUC values 0.84, 82.6% sensitivity and 88.9% specificity; Fig. 6a), whereas the diagnostic values for miR-221-3p were lower (AUC values 0.64, 55.2% sensitivity and 80.0% specificity; Fig. 6b). Combining miR-133b with miR-221-3p increased the AUC values to 0.85 (84.8% sensitivity and 88.9% specificity; Fig. 6c). Interestingly, ROC curve analysis of miR-133b also showed good diagnostic value to distinguish MSA from controls (AUC values 0.88, 85.7% sensitivity and 84.4% specificity; Fig. 6d). The AUC value for miR-4454 to distinguish PD from MSA was 0.72with 57.1% sensitivity and 82.6% specificity (Fig. 6e). The diagnostic power of miRNAs to discriminate among PD, MSA and healthy controls is summarized in Table 5.

**Table 4:** Diagnostic power of miRNAs to discriminate among PD, MSA and healthy controls (measured by ROC curve analyses).

Groups	miRNAs	AUC	95% CI	Cut-off value	Sensitivity (%)	Specificity (%)
PD versus control	miR-133b	0.84	0.75–0.94	0.019	82.6	88.9
	miR-221-3p	0.64	0.53–0.76	0.33	55.2	80.0
	miR-133band miR-221-3p	0.85	0.77–0.94	0.40	84.8	88.9
MSA versus control	miR-133b	0.88	0.79–0.96	0.017	85.7	84.4
PD versus MSA	miR-4454	0.72	0.58–0.85	2.51	57.1	82.6
Early PD versus control	miR-133b	0.90	0.80–1.0	0.020	94.4	91.1
	miR-221-3p	0.67	0.51–0.83	0.36	55.6	80.0
	miR-133b and miR-221-3p	0.90	0.76–1.0	0.21	94.4	91.1

PD, Parkinson’s disease; MSA: multiple system atrophy; AUC, area under the curve; CI, confidence interval.

**Table 5:** Univariate and multivariate logistic regression analyses of candidate plasma miRNAs in predicting Parkinson’s disease from healthy controls.

MiRNAs	Univariate logistic				Multivariate logistic (stepwise)			
	*p *value	OR	95% CI		*p *value	OR	95% CI	
			Lower	Upper			Lower	Upper
miR-133b	< 0.001	2.74E+26	1.20E+13	6.27E+39	< 0.001	1.10E+34	4.85E+16	2.47E+51
miR-221-3p	0.012	5.70	1.47	22.02	0.041	1.17	0.60	1.52
miR-320a	0.07	1.34	0.98	1.85	Not selected			
miR-205	0.23	1.26E+42	0	1.66E+111	Not selected			
miR-627	0.37	1.28E+39	0	8.21E+124	Not selected			
miR-432	0.31	2.12E+34	0	1.45E+100	Not selected			
miR-4454	0.25	0.48	0.31	0.92	Not selected

#### Biomarkers for early PD diagnosis

**Figure 7:**
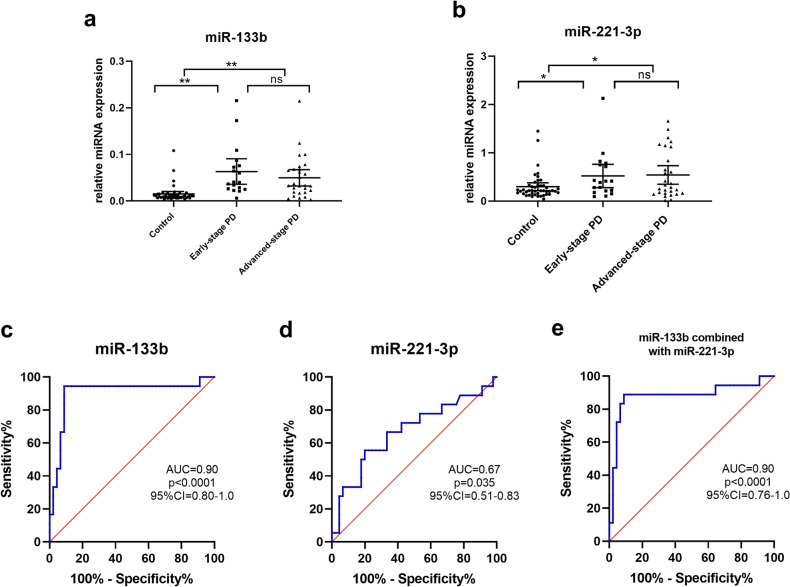
Plasma miR-133b (**a**) and miR-221-3p (**b**) expression in early-stage PD (modified H-Y stage 1–2.5, n = 18), advanced-stage PD (modified H-Y stage 3–5, n = 28) and healthy controls (n = 45) from cohort 3. ROC analysis of plasma miR-133b (**c**), miR-221-3p (**d**) and their combination (**e**) for discriminating patients with early-stage PD from healthy controls in cohort 3. The Kruskal–Wallis analysis with post hoc was used to evaluate differences between groups. Data are presented as the means ± SEMs. The results are indicated as **adjust *p* value < 0.01 and *adjust *p* value < 0.05. PD, Parkinson’s disease.

To identify biomarkers for early PD diagnosis, 18 PD cases in early clinical stages were selected for comparison with 45 controls in the validation set (Table 1). We also observed dramatical increases in the levels of miR-133b and miR-221-3p in early-stage PD compared to controls (for miR-133b, 4.21-fold increase, post-hoc of Kruskal–Wallis analysis, adjust *p* < 0.001; for miR-221-3p, 1.74-fold increase, post-hoc of Kruskal–Wallis analysis, adjust *p* = 0.035; Fig. 7a,b). The expression of seven candidate miRNAs in different PD stages and controls is shown in Fig. 7 and Supplementary Fig. 3.

For early PD diagnosis, the AUC values for miR-133b and miR-221-3p were 0.90 and 0.67, respectively. The sensitivity and specificity of miR-133b were 94.4% and 91.1%, respectively, and those of miR-221-3p were 55.6% and 80%, respectively (Fig. 7c,d). The combination of miR-133b and miR-221-3p did not change the diagnostic value (AUC value 0.90, 94.4% sensitivity and 91.1% specificity, Fig. 7e). The diagnostic power of miRNAs to discriminate between early-stage PD and healthy controls is summarized in Table 5.

## Discussion

Previous studies have identified several miRNAs that are potentially involved in the pathogenesis of PD^2,5,14^; however, the application of circulating miRNAs as biomarkers for PD remains debated^2,5^. Blood and its derivatives are the most extensively studied sources of biomarkers. Previous studies have indicated that plasma is more suitable than serum in studying circulating miRNA, as RNA released during the coagulation process may change the true repertoire of circulating miRNA^10^. However, plasma miRNAs are present at lower concentrations than serum miRNAs, thereby necessitating higher requirements for detection methods. In this study, we measured plasma circulating miRNA expression in three independent sets with a total of 151 PD, 21 MSA and 138 healthy controls using high-throughput RT-PCR. We identified elevated miR-133b and miR-221-3p levels that differentiated early-stage PD from controls with good diagnostic value. Moreover, we confirmed that a combination of miR-133b, miR-221-3p and miR-4454 distinguished PD from controls and MSA.

L-Dopa and related PD medications have been shown to dramatically affect the expression of certain miRNAs in PD^19^. In this study, we did not find any difference in the expression of seven candidate miRNAs between the drug-treated group and drug-naïve group, minimizing the contamination of miRNA expression by drug treatment in the following studies. Sex-based differences of miRNAs have been reported in human brains as well as in peripheral blood component from literatures, including miR-320 and miR-221^20,21^. In this study, we compared seven candidate miRNAs expression between male and female in both duplication set and validation set, and failed to find stable sex-based difference. In addition, analyses of covariance between groups with sex and age as covariates showed sex and age did not exert significant effects on the considered miRNAs, minimizing gender-induced bias. The next comparison between PD and controls showed remarkable upregulation of plasma miR-133b, miR-221-3p and miR-320a in PD, and elevated miR-133b and miR-221-3p levels discriminated early-stage PD from controls with good diagnostic value. MiR-133b is one of the first reported miRNAs playing roles in the maintenance of midbrain dopaminergic neurons in both cell and animal models^13^. MiR-133b is specifically enriched in the midbrain and deficient in PD compared to the control^13^. Reduced serum circulating miR-133b has also been observed in PD patients compared to controls^22,23^. Interestingly, high-throughput small RNA sequencing in a *Drosophila* PD model presents consistent upregulation of miR-133 in early-stage PD files^24^. The precise pathophysiological functions of miR-133b in PD are not completely understood. Overexpression of miR-133b in primary embryonic rat midbrain culture reduced dopaminergic neuron numbers and dopamine release, whereas its suppression had the opposite effect, suggesting that it could be a negative regulator of dopaminergic neurons^13^. Hence, the simplest explanation for miR-133b deficiency in the PD brain and its toxic mouse model is the loss of dopamine neurons where miR-133b is normally enriched^25^. In this study, we identified significant upregulation of plasma circulating miR-133b expression in both the early-stage and advanced-stage PD groups independent of L-dopa treatment. These findings are consistent with the limited effect of PD drug treatment on miR-133b expression^19^. Surprisingly, miR-133b was also upregulated in MSA compared to controls, suggesting that miR-133b may also be related to α-synuclein pathologies in addition to dopaminergic neurons. A recent report inferred that miR-133b indirectly influences α-synuclein by targeting the Ras homolog gene family member A (RhoA)^26^. MiR-221-3p is another upregulated miRNA identified in PD in this study. To our knowledge, at least five previous studies have assessed miR-221 expression in PD (Table 3)^27–31^. Interestingly, miR-221 expression in brain tissue is remarkably increased in PD^27,28^, and elevated cingulate miR-221 correlates with SNCA and PARK2 transcript levels^28^. However, the alterations in serum miR-221 levels in PD are variable in different studies^29–31^. These discrepant results from brain tissue and serum have not yet been completely explained. MiR‐221 is highly expressed in glioma cells and implicated in various biological processes, including apoptosis, cell cycle and differentiation^32^. Cytobiological studies have shown that miR-221 may be involved in the pathogenesis of PD by regulating autophagy^33^, oxidative pathways^34^and iron uptake^35^. To date, there has been no assessment of circulating plasma miR-221 expression in PD. In the present work, we performed triplicate measurements in three independent sets and identified that elevated plasma miR-221-3p predicts both early and advanced PD in controls. In addition, increased plasma miR-221-3p expression was only observed in PD but not in MSA compared to controls, indicating a potential differentiation value of miR-221-3p between PD and MSA. Previous studies have shown that miR-320a inhibits cell proliferation and regulates tumor occurrence, progression and metastasis. MiR-320a is decreased in the blood samples of colorectal cancer^36^ and increased in the sera of patients with Alzheimer’s disease(AD)^37^. Interestingly, a previous study also showed a reduction in miR-320a in blood leukocytes of PD patients compared to controls^15^. In this study, we observed the upregulation of plasma miR-320a in the advanced stage of PD compared to healthy controls in duplication set. However, the increases of miR-320a in PD group could not be replicated in the validation set. The underlying mechanism by which miR-320a contributes to neurodegeneration remains to be investigated. Overexpression of the miR-320a mimic has been shown to precipitate α-synuclein accumulation by suppressing heat shock protein 70^16^. Combined with findings of altered miR-320a expression in AD^37^, PD and MSA, we propose that miR-320a may have a close relationship to synucleinopathies and be involved later in disease progression than miR-133b. But the instable alternations of miR-320a in duplication set and validation set in this study doubt its real role in PD and more studies with larger sample size are needed to identify this result. MiR-4454 is a relatively new microRNA with limited investigation. Most current studies of miR-4454 focus on cancers^38^, and no qualification of miR-4454 expression in PD has been reported. In the present study, miR-4454 levels were unchanged in global PD compared to controls in both the duplication set and validation set. However, plasma miR-4454 levels in PD patients were lower than those in MSA patients, suggesting that miR-4454 could be a potential supplementary biomarker for differentiating PD from other neurodegenerative disorders.

Previous studies investigating the expression of miR-133b, miR-221 and miR-320a in various tissues of PD are summarized in Table 6. We noticed there are some discrepancies. This difference may be explained by several reasons. First, different sample sources and measurement methods from different studies may lead to conflicting results. Second, since miRNAs are tissue- and disease-specific, there is a lack of universally accepted reference miRNAs to which target miRNAs can be normalized. U6 RNA and 5S rRNA, which are normally used as housekeeping miRNAs for tissue miRNA normalization, are degraded in serum samples^29^. Finally, the storage time of samples should also be considered^39^.

**Table 6:** Summary of three miRNA (miR-133b, miR-221 and miR-320a) alterations in Parkinson’s disease compared to controls from previous studies.

miRNAs	Expression alternation	Sample sizes	Source	References
miR-133b	Reduced	3 PD, 5 controls	Brain	^13^
	Reduced	46 PD, 46 controls	Serum	^22^
	Reduced	46 PD, 49 controls	Serum	^23^
	Not significant	138 PD, 112 controls	Serum	^30^
	Not significant	20 untreated PD and18 treated PD, 24 controls	PBLCs	^19^
	Not significant	28 PD, 28 controls	CSF	^43^
miR-221	Increased	22 PD, 10 controls	Brain	^28^
	Increased	12 PD, 12 controls	Brain	^27^
	Reduced	138 PD, 112 controls	Serum	^30^
	Reduced	106 PD, 91 controls	Serum	^29^
	Not significant	51 PD, 20 controls	Serum	^31^
miR-320a	Reduced	7PD, 6 controls	PBLCs	^15^

PBLCs, peripheral blood lymphocytes; CSF, cerebrospinal fluid; PD, Parkinson’s disease.

There are a few limitations in this study. First, all samples in the current study were from mono-center in China, and further international multiple center studies are needed to avoid selection and racial bias. Second, the alternations of miR-320a in duplication set and validation set were inconsistent, showed the challenges of miR-320a as a biomarker for PD diagnosis. Third, only limited MSA samples were assessed as disease controls in this study. More neurodegenerative diseases are needed to verify the diagnostic value of the detected miRNAs in the future.

In summary, this work revealed that upregulated plasma miR-133b and miR-221-3p potentially represent good biomarkers for early PD, and a combination of miR-133b, miR-221-3p and miR-4454 has the potential to serve as a noninvasive biomarker for PD diagnosis.

## Materials and methods

### Participants and experiments design

The study was approved by the Ethics Committee of Xiangya Hospital (2013Q09). All individuals were informed of the use of blood samples and signed a written informed consent before the start of the study. A total of 310 participants with 151 PD, 21 MSA and 138 healthy controls were recruited in this investigation for three independent test sets. All PD patients met the diagnostic criteria of the Movement Disorder Society in 2015^40^, and all MSA patients were diagnosed according to Gilman’s criteria^41^. In addition, all PD and MSA cases were confirmed by two independent senior neurologists. Participants with other diseases, such as cancer, diabetes, cardiovascular disorders, infection and any other neurological or psychiatric disorders, were excluded.

In the initial screening set, 78 PD cases and 78 healthy controls were randomly divided into 6 groups (26 samples of each group) for plasma miRNA profiling. In the duplication test set, 27 PD cases (including 13 L-dopa-naïve PD and 14 L-dopa-treated PD) and 15 healthy controls were included for candidate miRNA assessments. In the validation set, 46 PD, 21 MSA and 45 healthy controls were recruited.

### Sample collection and circulating RNA extraction

Five milliliters of peripheral whole blood were collected from each participant in EDTA anticoagulation tubes and kept at room temperature for 1 h. Then, blood samples were centrifuged at 3000 rpm and 4 °C for 15 min. The supernatants were plasma, aliquoted and stored at − 80 °C. Circulating RNAs were isolated from plasma by the miRsol method as described in our previous study^11^. Caenorhabditis elegans cel-miR-54-5p (0.1 pM) was added as a normalization control. MiR-451a and U6 RNA were used as quality controls to exclude cell and hemolysis contamination.

### Polyadenylation, reverse transcription

The polyadenylation and reverse transcription procedure were conducted exactly according to the S-Poly(T) Plus protocol^11,42^. To profile miRNAs effectively, every 7 out of 486 miRNAs as well as spiked-in cel-miR-54 were grouped for the one-step reaction of polyadenylation and reverse transcription simultaneously. MiRNAs with identical forward primers or with more than five base pairings between the forward primer and RT primers were avoided in the same group. All sequences, primers and probes are listed in Supplemental Table 1.

### Quantitative real-time PCR

qRT-PCR was performed in 96-well plates by using an ABI Step-One Plus thermal cycler as previously described^11^. Each PCR was performed in duplicate. The miRNA expression level was normalized to spiked-in cel-miR-54-5p. MiRNAs with cycle threshold (Ct) values less than 35 in the panel were included in the data analysis.

### Statistical

All analyses were performed with SPSS 22.0 (SPSS Inc., Chicago, IL, USA). Data were presented as the mean ± SEM. A paired t-test was used to identify candidate miRNAs in the screening step. Independent t-tests were used to assess differences between groups in the duplication set, and nonparametric analysis of the Mann–Whitney U test was used to assess differences between groups in the validation set. Univariate and multivariate logistic regression analyses were used to assess the diagnostic value of candidate miRNAs.

A receiver operating characteristic (ROC) curve was used to calculate the relationship between sensitivity and specificity for the disease group versus healthy or disease controls and hence evaluate the diagnostic performance of the analyses, either individually or in combination. The “optimum” cutoff value from the ROC curve was determined by the sum of sensitivity, and specificity was maximal. *p* value < 0.05 were considered to be significant.

### Informed consent

Informed consent was obtained from all subjects involved in the study.

### Ethical approval

All procedures performed in studies involving human participants were in accordance with the ethical standards of the ethical committee of the Xiangya Hospital, Central South University, China, and with the 1964 Helsinki declaration and its later amendments or comparable ethical standards.

## Supplementary Information

Supplementary Information 1.

Supplementary Information 2.

Supplementary Information 3.

Supplementary Information 4.

Supplementary Information 5.

## Data Availability

The data that support the findings of this study are available from the corresponding author upon reasonable request.

## Abbreviations

AD: Alzheimer’s disease
AUC: Area under the curve
CSF: Cerebral spinal fluid
H-Y: Hoehn and Yahn
miRNAs: MicroRNAs
MSA: Multiple system atrophy
PD: Parkinson’s disease
qRT-PCR: Quantitative real-time PCR
RhoA: Ras homolog gene family member A
ROC: Receiver operating characteristic
