# Supplementary figures and images for: Elevated plasma miR-133b and miR-221-3p as biomarkers for early Parkinson’s disease
Source: Sci Rep. 2021 Jul 27;11:15268. doi: 10.1038/s41598-021-94734-z (PMC8316346; doi:10.1038/s41598-021-94734-z)

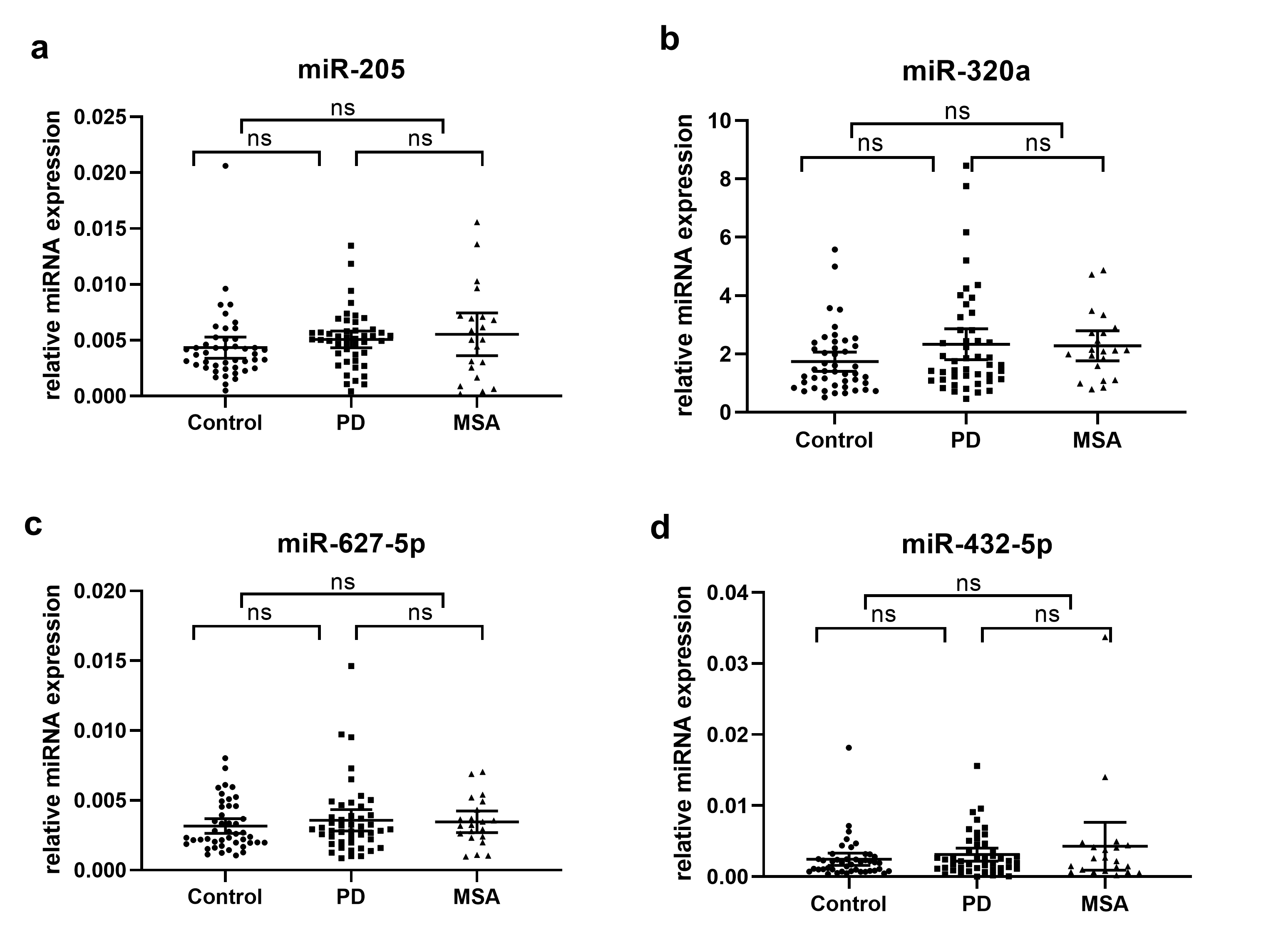

Supplement: Supplementary file 1 — Supplementary Information 1. [file 41598_2021_94734_MOESM1_ESM.png]

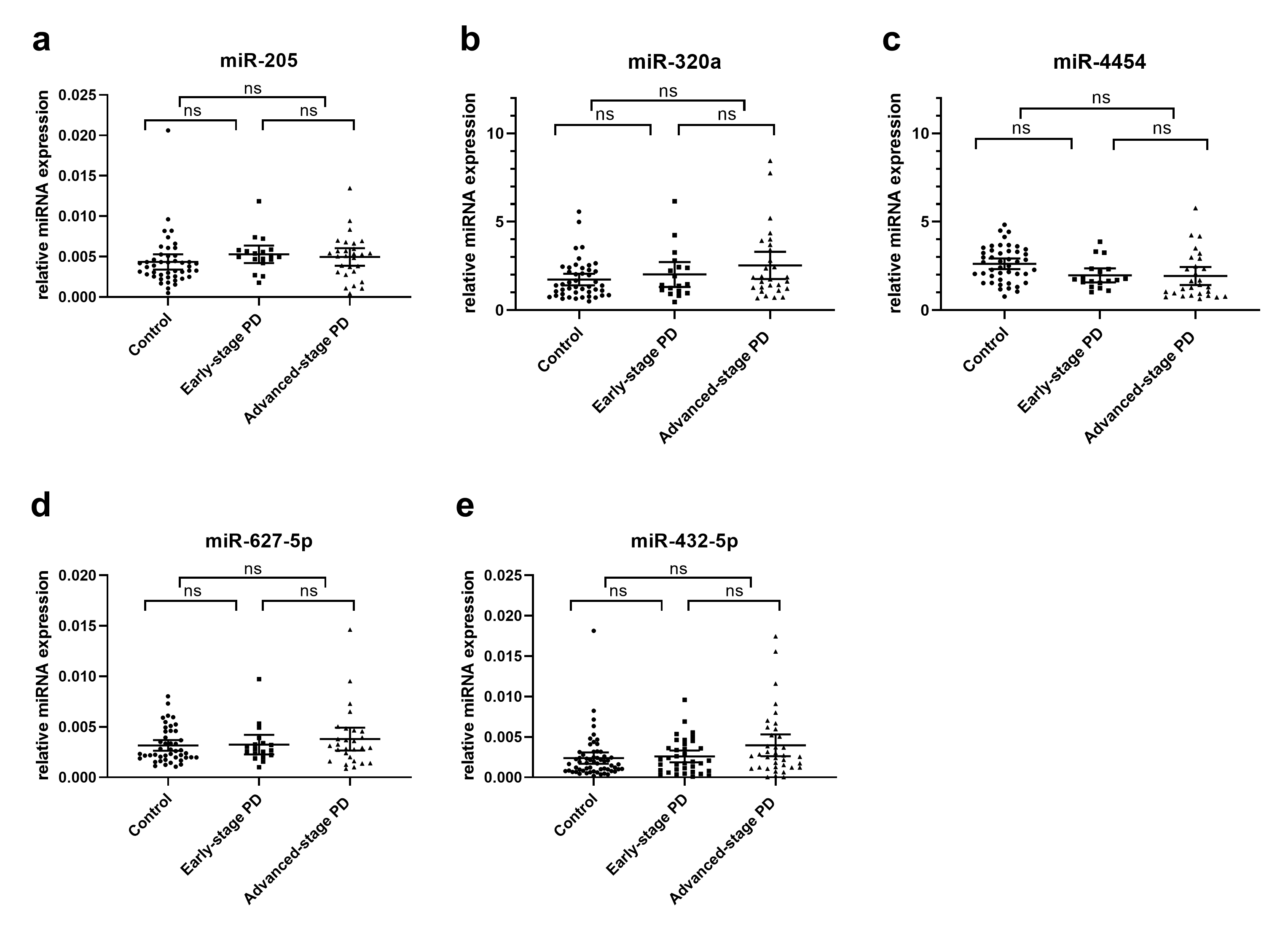

Supplement: Supplementary file 2 — Supplementary Information 2. [file 41598_2021_94734_MOESM2_ESM.png]

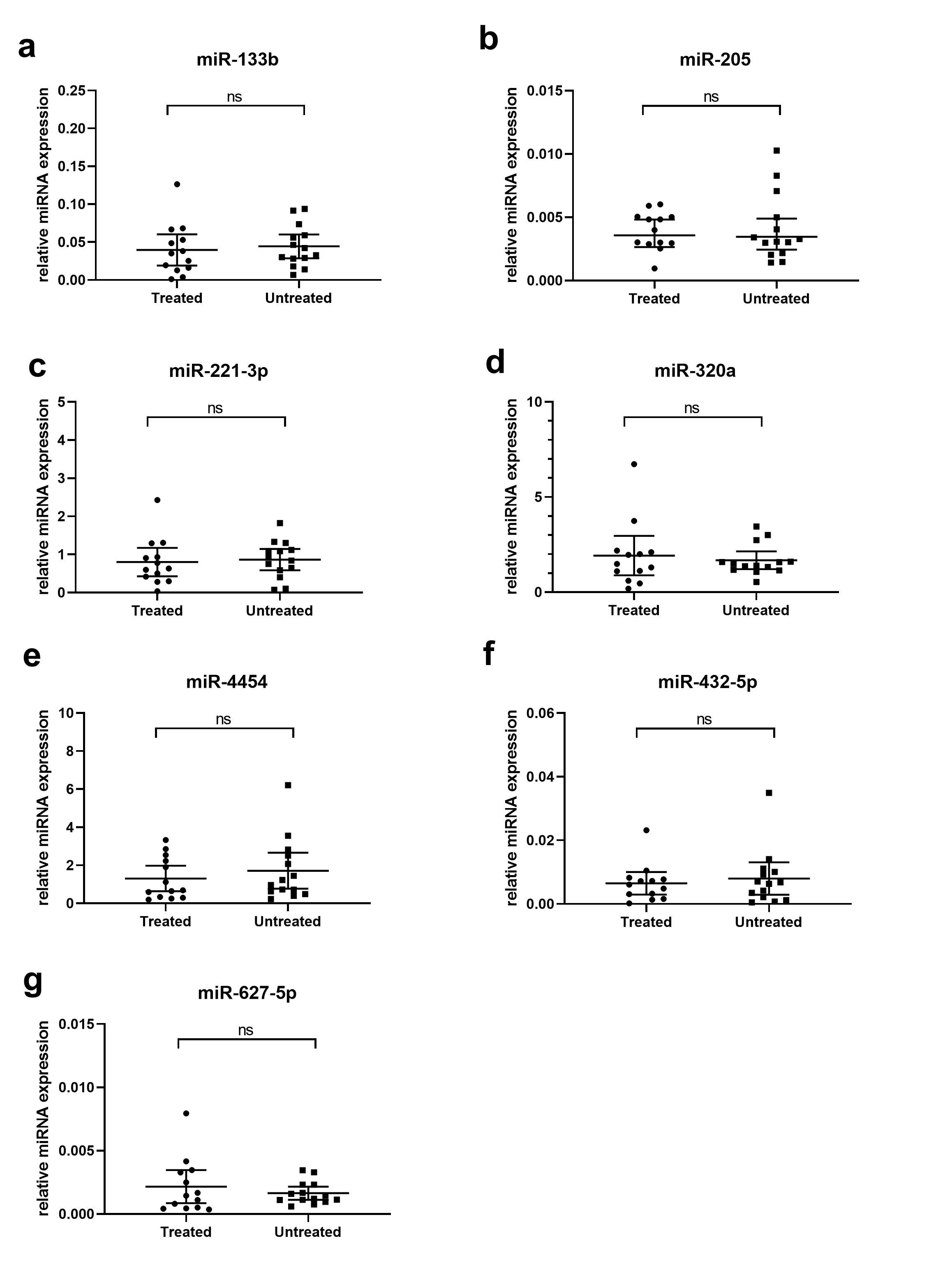

Supplement: Supplementary file 3 — Supplementary Information 3. [file 41598_2021_94734_MOESM3_ESM.jpg]
