# Supplementary material for: Elevated plasma miR-133b and miR-221-3p as biomarkers for early Parkinson’s disease
Source: Sci Rep. 2021 Jul 27;11:15268. doi: 10.1038/s41598-021-94734-z (PMC8316346; doi:10.1038/s41598-021-94734-z)
Supplement: Supplementary file 4 — Supplementary Information 4. [file 41598_2021_94734_MOESM4_ESM.docx]

**Supplementary figure legends**

Figure S1: No statistically significant differences were found in the levels of seven candidate miRNAs between the L-dopa-naïve (n=13) and L-dopa-treated Parkinson’s disease groups (n=14) in cohort 2 (a-g). An independent t-test was used to evaluate differences between groups. Data are presented as the means ± SEMs. ns, no significance.

Figure S2: No significant differences were found in the expression of miR-205 (a), miR-320a(b), miR-627-5p (c) or miR-432-5p (d) among patients with PD (n=46), patients with MSA (n=21) and controls (n=45) in cohort 3. The Kruskal-Wallis analysis with post hoc was used to evaluate differences between groups. Data are presented as the means ± SEMs. PD, Parkinson’s disease; MSA, multiple system atrophy; ns, no significance.

Figure S3: No significant differences in the expression of miR-205 (a), miR-320a(b), miR-4454 (c), miR-627-5p (d) or miR-432-5p (e) were found among patients with early-stage PD (modified H-Y stage 1-2.5, n=18), patients with advanced-stage PD (modified H-Y stage 3-5, n=28) and controls (n=45) in cohort 3. The Kruskal-Wallis analysis with post hoc was used to evaluate differences between groups. Data are presented as the means ± SEMs. PD, Parkinson’s disease; ns, no significance.
