# Supplementary material for: Elevated plasma miR-133b and miR-221-3p as biomarkers for early Parkinson’s disease
Source: Sci Rep. 2021 Jul 27;11:15268. doi: 10.1038/s41598-021-94734-z (PMC8316346; doi:10.1038/s41598-021-94734-z)
Supplement: Supplementary file 5 — Supplementary Information 5. [file 41598_2021_94734_MOESM5_ESM.docx]

Supplementary table 1: Primers and probe sequences for 486 miRNAs

| Group in RT | miRNA | Forward primer | Sequence (5’-3’) | RT-primer |
| --- | --- | --- | --- | --- |
| 1 | >hsa-miR-215-5p MIMAT0000272 | TGTCGGATGACCTATGAATTG | AUGACCUAUGAAUUGACAGAC | GTGCAGGGTCCGAGGTCAGAGCCACCTGGGCAATTTTTTTTTTTGTCTGT |
| 1 | >hsa-miR-2355-3p MIMAT0017950 | TTCGGATTGTCCTTGCTGTTT | AUUGUCCUUGCUGUUUGGAGAU | GTGCAGGGTCCGAGGTCAGAGCCACCTGGGCAATTTTTTTTTTTATCTCC |
| 1 | >hsa-miR-3146 MIMAT0015018 | TTCGGCATGCTAGGATAGAAA | CAUGCUAGGAUAGAAAGAAUGG | GTGCAGGGTCCGAGGTCAGAGCCACCTGGGCAATTTTTTTTTTTCCATTC |
| 1 | >hsa-miR-29b-3p MIMAT0000100 | TCGGTAGCACCATTTGAAATC | UAGCACCAUUUGAAAUCAGUGUU | GTGCAGGGTCCGAGGTCAGAGCCACCTGGGCAATTTTTTTTTTTAACACT |
| 1 | >hsa-miR-153-3p MIMAT0000439 | GTCGGTTGCATAGTCACAAAA | UUGCAUAGUCACAAAAGUGAUC | GTGCAGGGTCCGAGGTCAGAGCCACCTGGGCAATTTTTTTTTTTGATCAC |
| 1 | >hsa-miR-15a-3p MIMAT0004488 | CGGCAGGCCATATTGTGCT | CAGGCCAUAUUGUGCUGCCUCA | GTGCAGGGTCCGAGGTCAGAGCCACCTGGGCAATTTTTTTTTTTGAGGCA |
| 1 | >hsa-miR-1307-5p MIMAT0022727 | TGGTCGACCGGACCTCGA | UCGACCGGACCUCGACCGGCU | GTGCAGGGTCCGAGGTCAGAGCCACCTGGGCAATTTTTTTTTTTAGCCGG |
| 2 | >hsa-miR-221-5p MIMAT0004568 | TCGGACCTGGCATACAATGT | ACCUGGCAUACAAUGUAGAUUU | GTGCAGGGTCCGAGGTCAGAGCCACCTGGGCAATTTTTTTTTTTAAATCT |
| 2 | >hsa-miR-502-5p MIMAT0002873 | TTCGGATCCTTGCTATCTGG | AUCCUUGCUAUCUGGGUGCUA | GTGCAGGGTCCGAGGTCAGAGCCACCTGGGCAATTTTTTTTTTTAGCACC |
| 2 | >hsa-let-7b-3p MIMAT0004482 | TCGGCTATACAACCTACTGC | CUAUACAACCUACUGCCUUCCC | GTGCAGGGTCCGAGGTCAGAGCCACCTGGGCAATTTTTTTTTTTGGGAAG |
| 2 | >hsa-miR-199b-5p MIMAT0000263 | CGGCCCAGTGTTTAGACTAT | CCCAGUGUUUAGACUAUCUGUUC | GTGCAGGGTCCGAGGTCAGAGCCACCTGGGCAATTTTTTTTTTTGAACAG |
| 2 | >hsa-miR-378h MIMAT0018984 | TCGGACTGGACTTGGTGTC | ACUGGACUUGGUGUCAGAUGG | GTGCAGGGTCCGAGGTCAGAGCCACCTGGGCAATTTTTTTTTTTCCATCT |
| 2 | >hsa-miR-3169 MIMAT0015044 | TGGTAGGACTGTGCTTGGC | UAGGACUGUGCUUGGCACAUAG | GTGCAGGGTCCGAGGTCAGAGCCACCTGGGCAATTTTTTTTTTTCTATGT |
| 2 | >hsa-miR-376b MIMAT0002172 | GTCGGATCATAGAGGAAAATC | AUCAUAGAGGAAAAUCCAUGUU | GTGCAGGGTCCGAGGTCAGAGCCACCTGGGCAATTTTTTTTTTTAACATG |
| 3 | >hsa-miR-1915-3p MIMAT0007892 | GGCCCCAGGGCGACGC | CCCCAGGGCGACGCGGCGGG | GTGCAGGGTCCGAGGTCAGAGCCACCTGGGCAATTTTTTTTTTTCCCGCC |
| 3 | >hsa-miR-17-3p MIMAT0000071 | TGGACTGCAGTGAAGGCAC | ACUGCAGUGAAGGCACUUGUAG | GTGCAGGGTCCGAGGTCAGAGCCACCTGGGCAATTTTTTTTTTTCTACAA |
| 3 | >hsa-miR-199b-3p MIMAT0004563 | TCGGACAGTAGTCTGCACAT | ACAGUAGUCUGCACAUUGGUUA | GTGCAGGGTCCGAGGTCAGAGCCACCTGGGCAATTTTTTTTTTTAACCAA |
| 3 | >hsa-miR-142-3p MIMAT0000434 | TCGGTGTAGTGTTTCCTACTT | UGUAGUGUUUCCUACUUUAUGGA | GTGCAGGGTCCGAGGTCAGAGCCACCTGGGCAATTTTTTTTTTTCCATAA |
| 3 | >hsa-miR-3653-3p MIMAT0018073 | TCGGTCGGCTAAGAAGTTGA | CUAAGAAGUUGACUGAAG | GTGCAGGGTCCGAGGTCAGAGCCACCTGGGCAATTTTTTTTTTTCTTCAG |
| 3 | >hsa-miR-3164 MIMAT0015038 | TTCGGTGTGACTTTAAGGGAA | UGUGACUUUAAGGGAAAUGGCG | GTGCAGGGTCCGAGGTCAGAGCCACCTGGGCAATTTTTTTTTTTCGCCAT |
| 3 | >hsa-miR-27b-3p MIMAT0000419 | TTCGGTTCACAGTGGCTAAG | UUCACAGUGGCUAAGUUCUGC | GTGCAGGGTCCGAGGTCAGAGCCACCTGGGCAATTTTTTTTTTTGCAGAA |
| 4 | >hsa-miR-223-5p MIMAT0004570 | TCGGCGTGTATTTGACAAGC | CGUGUAUUUGACAAGCUGAGUU | GTGCAGGGTCCGAGGTCAGAGCCACCTGGGCAATTTTTTTTTTTAACTCA |
| 4 | >hsa-miR-486-3p MIMAT0004762 | TGGCGGGGCAGCTCAGTA | CGGGGCAGCUCAGUACAGGAU | GTGCAGGGTCCGAGGTCAGAGCCACCTGGGCAATTTTTTTTTTTATCCTG |
| 4 | >hsa-miR-496 MIMAT0002818 | TCGGTGAGTATTACATGGCC | UGAGUAUUACAUGGCCAAUCUC | GTGCAGGGTCCGAGGTCAGAGCCACCTGGGCAATTTTTTTTTTTGAGATT |
| 4 | >hsa-miR-493-3p MIMAT0003161 | TCGGTGAAGGTCTACTGTGT | UGAAGGUCUACUGUGUGCCAGG | GTGCAGGGTCCGAGGTCAGAGCCACCTGGGCAATTTTTTTTTTTCCTGGC |
| 4 | >hsa-let-7a-5p MIMAT0000062 | TCGGTGAGGTAGTAGGTTGT | UGAGGUAGUAGGUUGUAUAGUU | GTGCAGGGTCCGAGGTCAGAGCCACCTGGGCAATTTTTTTTTTTAACTAT |
| 4 | >hsa-miR-361-3p MIMAT0004682 | TGTCCCCCAGGTGTGATTC | UCCCCCAGGUGUGAUUCUGAUUU | GTGCAGGGTCCGAGGTCAGAGCCACCTGGGCAATTTTTTTTTTTAAATCA |
| 4 | >hsa-miR-3148 MIMAT0015021 | TTCGGTGGAAAAAACTGGTGT | UGGAAAAAACUGGUGUGUGCUU | GTGCAGGGTCCGAGGTCAGAGCCACCTGGGCAATTTTTTTTTTTAAGCAC |
| 5 | >hsa-miR-15b-3p MIMAT0004586 | GTCGGCGAATCATTATTTGCT | CGAAUCAUUAUUUGCUGCUCUA | GTGCAGGGTCCGAGGTCAGAGCCACCTGGGCAATTTTTTTTTTTAGAGCA |
| 5 | >hsa-miR-609 MIMAT0003277 | GTCGGAGGGTGTTTCTCTC | AGGGUGUUUCUCUCAUCUCU | GTGCAGGGTCCGAGGTCAGAGCCACCTGGGCAATTTTTTTTTTTAGAGAT |
| 5 | >hsa-miR-584-3p MIMAT0022708 | TGGTCAGTTCCAGGCCAAC | UCAGUUCCAGGCCAACCAGGCU | GTGCAGGGTCCGAGGTCAGAGCCACCTGGGCAATTTTTTTTTTTAGCCTG |
| 5 | >hsa-miR-154-5p MIMAT0000452 | TCGGTAGGTTATCCGTGTTG | UAGGUUAUCCGUGUUGCCUUCG | GTGCAGGGTCCGAGGTCAGAGCCACCTGGGCAATTTTTTTTTTTCGAAGG |
| 5 | >hsa-miR-584-5p MIMAT0003249 | CGGTTATGGTTTGCCTGGG | UUAUGGUUUGCCUGGGACUGAG | GTGCAGGGTCCGAGGTCAGAGCCACCTGGGCAATTTTTTTTTTTCTCAGT |
| 5 | >hsa-miR-1226-3p MIMAT0005577 | TGGTCACCAGCCCTGTGTT | UCACCAGCCCUGUGUUCCCUAG | GTGCAGGGTCCGAGGTCAGAGCCACCTGGGCAATTTTTTTTTTTCTAGGG |
| 5 | >hsa-miR-519a-5p MIMAT0005452 | GGCTCTAGAGGGAAGCGC | CUCUAGAGGGAAGCGCUUUCUG | GTGCAGGGTCCGAGGTCAGAGCCACCTGGGCAATTTTTTTTTTTCAGAAA |
| 6 | >hsa-miR-196b-3p MIMAT0009201 | TGGTCGACAGCACGACACT | UCGACAGCACGACACUGCCUUC | GTGCAGGGTCCGAGGTCAGAGCCACCTGGGCAATTTTTTTTTTTGAAGGC |
| 6 | >hsa-miR-579 MIMAT0003244 | TTCGGTTCATTTGGTATAAACC | UUCAUUUGGUAUAAACCGCGAUU | GTGCAGGGTCCGAGGTCAGAGCCACCTGGGCAATTTTTTTTTTTAATCGC |
| 6 | >hsa-miR-126-3p MIMAT0000445 | TCGGTCGTACCGTGAGTAAT | UCGUACCGUGAGUAAUAAUGCG | GTGCAGGGTCCGAGGTCAGAGCCACCTGGGCAATTTTTTTTTTTCGCATT |
| 6 | >hsa-miR-339-3p MIMAT0004702 | GTGAGCGCCTCGACGACA | UGAGCGCCUCGACGACAGAGCCG | GTGCAGGGTCCGAGGTCAGAGCCACCTGGGCAATTTTTTTTTTTCGGCTC |
| 6 | >hsa-miR-185-5p MIMAT0000455 | CGGTGGAGAGAAAGGCAGT | UGGAGAGAAAGGCAGUUCCUGA | GTGCAGGGTCCGAGGTCAGAGCCACCTGGGCAATTTTTTTTTTTCAGGAA |
| 6 | >hsa-miR-182-5p MIMAT0000259 | TGGTTTGGCAATGGTAGAACT | UUUGGCAAUGGUAGAACUCACACU | GTGCAGGGTCCGAGGTCAGAGCCACCTGGGCAATTTTTTTTTTTAGTGTG |
| 6 | >hsa-miR-30b-5p MIMAT0000420 | TTCGGTGTAAACATCCTACAC | UGUAAACAUCCUACACUCAGCU | GTGCAGGGTCCGAGGTCAGAGCCACCTGGGCAATTTTTTTTTTTAGCTGA |
| 7 | >hsa-miR-494-3p MIMAT0002816 | TCGGTGAAACATACACGGGA | UGAAACAUACACGGGAAACCUC | GTGCAGGGTCCGAGGTCAGAGCCACCTGGGCAATTTTTTTTTTTGAGGTT |
| 7 | >hsa-miR-551b-3p MIMAT0003233 | CGGGCGACCCATACTTGG | GCGACCCAUACUUGGUUUCAG | GTGCAGGGTCCGAGGTCAGAGCCACCTGGGCAATTTTTTTTTTTCTGAAA |
| 7 | >hsa-miR-23c MIMAT0018000 | TCGGATCACATTGCCAGTGA | AUCACAUUGCCAGUGAUUACCC | GTGCAGGGTCCGAGGTCAGAGCCACCTGGGCAATTTTTTTTTTTGGGTAA |
| 7 | >hsa-miR-34a-5p MIMAT0000255 | CGGTGGCAGTGTCTTAGCT | UGGCAGUGUCUUAGCUGGUUGU | GTGCAGGGTCCGAGGTCAGAGCCACCTGGGCAATTTTTTTTTTTACAACC |
| 7 | >hsa-miR-942-5p MIMAT0004985 | TCGGTCTTCTCTGTTTTGGC | UCUUCUCUGUUUUGGCCAUGUG | GTGCAGGGTCCGAGGTCAGAGCCACCTGGGCAATTTTTTTTTTTCACATG |
| 7 | >hsa-miR-520e MIMAT0002825 | TGTCGGAAAGTGCTTCCTTTT | AAAGUGCUUCCUUUUUGAGGG | GTGCAGGGTCCGAGGTCAGAGCCACCTGGGCAATTTTTTTTTTTCCCTCA |
| 7 | >hsa-miR-340-5p MIMAT0004692 | TGTCGGTTATAAAGCAATGAGA | UUAUAAAGCAAUGAGACUGAUU | GTGCAGGGTCCGAGGTCAGAGCCACCTGGGCAATTTTTTTTTTTAATCAG |
| 8 | >hsa-miR-200a-3p MIMAT0000682 | TTCGGTAACACTGTCTGGTAA | UAACACUGUCUGGUAACGAUGU | GTGCAGGGTCCGAGGTCAGAGCCACCTGGGCAATTTTTTTTTTTACATCG |
| 8 | >hsa-miR-138-2-3p MIMAT0004596 | CGGGCTATTTCACGACACC | GCUAUUUCACGACACCAGGGUU | GTGCAGGGTCCGAGGTCAGAGCCACCTGGGCAATTTTTTTTTTTAACCCT |
| 8 | >hsa-miR-1539 MIMAT0007401 | GGTCCTGCGCGTCCCAG | UCCUGCGCGUCCCAGAUGCCC | GTGCAGGGTCCGAGGTCAGAGCCACCTGGGCAATTTTTTTTTTTGGGCAT |
| 8 | >hsa-miR-449c-3p MIMAT0013771 | TGGTTGCTAGTTGCACTCCT | UUGCUAGUUGCACUCCUCUCUGU | GTGCAGGGTCCGAGGTCAGAGCCACCTGGGCAATTTTTTTTTTTACAGAG |
| 8 | >hsa-miR-4286 MIMAT0016916 | TGGTCGGACCCCACTCCT | ACCCCACUCCUGGUACC | GTGCAGGGTCCGAGGTCAGAGCCACCTGGGCAATTTTTTTTTTTGGTACC |
| 8 | >hsa-miR-519a-3p MIMAT0002869 | GTCGGAAAGTGCATCCTTTTA | AAAGUGCAUCCUUUUAGAGUGU | GTGCAGGGTCCGAGGTCAGAGCCACCTGGGCAATTTTTTTTTTTACACTC |
| 8 | >hsa-miR-1251 MIMAT0005903 | TTCGGACTCTAGCTGCCAAA | ACUCUAGCUGCCAAAGGCGCU | GTGCAGGGTCCGAGGTCAGAGCCACCTGGGCAATTTTTTTTTTTAGCGCC |
| 9 | >hsa-miR-4324 MIMAT0016876 | TTCGGCCCTGAGACCCTAA | CCCUGAGACCCUAACCUUAA | GTGCAGGGTCCGAGGTCAGAGCCACCTGGGCAATTTTTTTTTTTAAGGTT |
| 9 | >hsa-miR-582-3p MIMAT0004797 | TTCGGTAACTGGTTGAACAAC | UAACUGGUUGAACAACUGAACC | GTGCAGGGTCCGAGGTCAGAGCCACCTGGGCAATTTTTTTTTTTGGTTCA |
| 9 | >hsa-miR-627-5p MIMAT0003296 | TTCGGGTGAGTCTCTAAGAAA | GUGAGUCUCUAAGAAAAGAGGA | GTGCAGGGTCCGAGGTCAGAGCCACCTGGGCAATTTTTTTTTTTCCTCTT |
| 9 | >hsa-miR-30a-5p MIMAT0000087 | TCGGTGTAAACATCCTCGAC | UGUAAACAUCCUCGACUGGAAG | GTGCAGGGTCCGAGGTCAGAGCCACCTGGGCAATTTTTTTTTTTCTTCCA |
| 9 | >hsa-miR-184 MIMAT0000454 | CGGTGGACGGAGAACTGAT | UGGACGGAGAACUGAUAAGGGU | GTGCAGGGTCCGAGGTCAGAGCCACCTGGGCAATTTTTTTTTTTACCCTT |
| 9 | >hsa-miR-20b-3p MIMAT0004752 | CGGACTGTAGTATGGGCAC | ACUGUAGUAUGGGCACUUCCAG | GTGCAGGGTCCGAGGTCAGAGCCACCTGGGCAATTTTTTTTTTTCTGGAA |
| 9 | >hsa-miR-3925-5p MIMAT0018200 | TTCGGAAGAGAACTGAAAGTG | AAGAGAACUGAAAGUGGAGCCU | GTGCAGGGTCCGAGGTCAGAGCCACCTGGGCAATTTTTTTTTTTAGGCTC |
| 10 | >hsa-miR-522-3p MIMAT0002868 | GTCGGAAAATGGTTCCCTTTA | AAAAUGGUUCCCUUUAGAGUGU | GTGCAGGGTCCGAGGTCAGAGCCACCTGGGCAATTTTTTTTTTTACACTC |
| 10 | >hsa-miR-423-5p MIMAT0004748 | GTGAGGGGCAGAGAGCGA | UGAGGGGCAGAGAGCGAGACUUU | GTGCAGGGTCCGAGGTCAGAGCCACCTGGGCAATTTTTTTTTTTAAAGTC |
| 10 | >hsa-miR-320b MIMAT0005792 | TCGGAAAAGCTGGGTTGAGA | AAAAGCUGGGUUGAGAGGGCAA | GTGCAGGGTCCGAGGTCAGAGCCACCTGGGCAATTTTTTTTTTTGCCCTC |
| 10 | >hsa-miR-532-5p MIMAT0002888 | CGGCATGCCTTGAGTGTAG | CAUGCCUUGAGUGUAGGACCGU | GTGCAGGGTCCGAGGTCAGAGCCACCTGGGCAATTTTTTTTTTTACGGTC |
| 10 | >hsa-miR-520g MIMAT0002858 | TGGACAAAGTGCTTCCCTTTA | ACAAAGUGCUUCCCUUUAGAGUGU | GTGCAGGGTCCGAGGTCAGAGCCACCTGGGCAATTTTTTTTTTTACACTC |
| 10 | >hsa-miR-583 MIMAT0003248 | TCGGCAAAGAGGAAGGTCC | CAAAGAGGAAGGUCCCAUUAC | GTGCAGGGTCCGAGGTCAGAGCCACCTGGGCAATTTTTTTTTTTGTAATG |
| 10 | >hsa-miR-885-5p MIMAT0004947 | TCGGTCCATTACACTACCCT | UCCAUUACACUACCCUGCCUCU | GTGCAGGGTCCGAGGTCAGAGCCACCTGGGCAATTTTTTTTTTTAGAGGC |
| 11 | >hsa-let-7a-3p MIMAT0004481 | TGTCGGCTATACAATCTACTG | CUAUACAAUCUACUGUCUUUC | GTGCAGGGTCCGAGGTCAGAGCCACCTGGGCAATTTTTTTTTTTGAAAGA |
| 11 | >hsa-miR-99b-5p MIMAT0000689 | GGCACCCGTAGAACCGAC | CACCCGUAGAACCGACCUUGCG | GTGCAGGGTCCGAGGTCAGAGCCACCTGGGCAATTTTTTTTTTTCGCAAG |
| 11 | >hsa-miR-3133 MIMAT0014998 | GGTCGGTAAAGAACTCTTAAAA | UAAAGAACUCUUAAAACCCAAU | GTGCAGGGTCCGAGGTCAGAGCCACCTGGGCAATTTTTTTTTTTATTGGG |
| 11 | >hsa-miR-92b-3p MIMAT0003218 | TGGTATTGCACTCGTCCCG | UAUUGCACUCGUCCCGGCCUCC | GTGCAGGGTCCGAGGTCAGAGCCACCTGGGCAATTTTTTTTTTTGGAGGC |
| 11 | >hsa-miR-324-5p MIMAT0000761 | GCGCATCCCCTAGGGCAT | CGCAUCCCCUAGGGCAUUGGUGU | GTGCAGGGTCCGAGGTCAGAGCCACCTGGGCAATTTTTTTTTTTACACCA |
| 11 | >hsa-miR-451a MIMAT0001631 | TTCGGAAACCGTTACCATTAC | AAACCGUUACCAUUACUGAGUU | GTGCAGGGTCCGAGGTCAGAGCCACCTGGGCAATTTTTTTTTTTAACTCA |
| 11 | >hsa-miR-2110 MIMAT0010133 | TGTTGGGGAAACGGCCGC | UUGGGGAAACGGCCGCUGAGUG | GTGCAGGGTCCGAGGTCAGAGCCACCTGGGCAATTTTTTTTTTTCACTCA |
| 12 | >hsa-miR-3610 MIMAT0017987 | GTCGGGAATCGGAAAGGAG | GAAUCGGAAAGGAGGCGCCG | GTGCAGGGTCCGAGGTCAGAGCCACCTGGGCAATTTTTTTTTTTCGGCGC |
| 12 | >hsa-miR-518c-3p MIMAT0002848 | CGGCAAAGCGCTTCTCTTTA | CAAAGCGCUUCUCUUUAGAGUGU | GTGCAGGGTCCGAGGTCAGAGCCACCTGGGCAATTTTTTTTTTTACACTC |
| 12 | >hsa-miR-410-3p MIMAT0002171 | GGTCGGAATATAACACAGATG | AAUAUAACACAGAUGGCCUGU | GTGCAGGGTCCGAGGTCAGAGCCACCTGGGCAATTTTTTTTTTTACAGGC |
| 12 | >hsa-miR-26a-5p MIMAT0000082 | TTCGGTTCAAGTAATCCAGGA | UUCAAGUAAUCCAGGAUAGGCU | GTGCAGGGTCCGAGGTCAGAGCCACCTGGGCAATTTTTTTTTTTAGCCTA |
| 12 | >hsa-miR-193b-3p MIMAT0002819 | CGGAACTGGCCCTCAAAGT | AACUGGCCCUCAAAGUCCCGCU | GTGCAGGGTCCGAGGTCAGAGCCACCTGGGCAATTTTTTTTTTTAGCGGG |
| 12 | >hsa-miR-1538 MIMAT0007400 | CGGCCCGGGCTGCTGCT | CGGCCCGGGCUGCUGCUGUUCCU | GTGCAGGGTCCGAGGTCAGAGCCACCTGGGCAATTTTTTTTTTTAGGAAC |
| 12 | >hsa-miR-181b-5p MIMAT0000257 | CGGAACATTCATTGCTGTCG | AACAUUCAUUGCUGUCGGUGGGU | GTGCAGGGTCCGAGGTCAGAGCCACCTGGGCAATTTTTTTTTTTACCCAC |
| 13 | >hsa-miR-30e-3p MIMAT0000693 | TCGGCTTTCAGTCGGATGTT | CUUUCAGUCGGAUGUUUACAGC | GTGCAGGGTCCGAGGTCAGAGCCACCTGGGCAATTTTTTTTTTTGCTGTA |
| 13 | >hsa-miR-1227 MIMAT0005580 | TTCGGCGTGCCACCCTTTT | CGUGCCACCCUUUUCCCCAG | GTGCAGGGTCCGAGGTCAGAGCCACCTGGGCAATTTTTTTTTTTCTGGGG |
| 13 | >hsa-miR-192-3p MIMAT0004543 | CGGCTGCCAATTCCATAGG | CUGCCAAUUCCAUAGGUCACAG | GTGCAGGGTCCGAGGTCAGAGCCACCTGGGCAATTTTTTTTTTTCTGTGA |
| 13 | >hsa-miR-381-3p MIMAT0000736 | TCGGTATACAAGGGCAAGCT | UAUACAAGGGCAAGCUCUCUGU | GTGCAGGGTCCGAGGTCAGAGCCACCTGGGCAATTTTTTTTTTTACAGAG |
| 13 | >hsa-miR-1271-5p MIMAT0005796 | TGGCTTGGCACCTAGCAAG | CUUGGCACCUAGCAAGCACUCA | GTGCAGGGTCCGAGGTCAGAGCCACCTGGGCAATTTTTTTTTTTGAGTGC |
| 13 | >hsa-miR-24-2-5p MIMAT0004497 | CGGTGCCTACTGAGCTGAA | UGCCUACUGAGCUGAAACACAG | GTGCAGGGTCCGAGGTCAGAGCCACCTGGGCAATTTTTTTTTTTCTGTGT |
| 13 | >hsa-miR-411-3p MIMAT0004813 | TCGGTATGTAACACGGTCCA | UAUGUAACACGGUCCACUAACC | GTGCAGGGTCCGAGGTCAGAGCCACCTGGGCAATTTTTTTTTTTGGTTAG |
| 14 | >hsa-miR-320e MIMAT0015072 | TGGTCGGAAAGCTGGGTTG | AAAGCUGGGUUGAGAAGG | GTGCAGGGTCCGAGGTCAGAGCCACCTGGGCAATTTTTTTTTTTCCTTCT |
| 14 | >hsa-miR-551b-5p MIMAT0004794 | CGGGAAATCAAGCGTGGGT | GAAAUCAAGCGUGGGUGAGACC | GTGCAGGGTCCGAGGTCAGAGCCACCTGGGCAATTTTTTTTTTTGGTCTC |
| 14 | >hsa-miR-181a-5p MIMAT0000256 | TGGAACATTCAACGCTGTCG | AACAUUCAACGCUGUCGGUGAGU | GTGCAGGGTCCGAGGTCAGAGCCACCTGGGCAATTTTTTTTTTTACTCAC |
| 14 | >hsa-miR-128-3p MIMAT0000424 | TCGGTCACAGTGAACCGGT | UCACAGUGAACCGGUCUCUUU | GTGCAGGGTCCGAGGTCAGAGCCACCTGGGCAATTTTTTTTTTTAAAGAG |
| 14 | >hsa-let-7d-3p MIMAT0004484 | TGGCTATACGACCTGCTGC | CUAUACGACCUGCUGCCUUUCU | GTGCAGGGTCCGAGGTCAGAGCCACCTGGGCAATTTTTTTTTTTAGAAAG |
| 14 | >hsa-miR-4301 MIMAT0016850 | GTCGGTCCCACTACTTCAC | UCCCACUACUUCACUUGUGA | GTGCAGGGTCCGAGGTCAGAGCCACCTGGGCAATTTTTTTTTTTCACAAG |
| 14 | >hsa-miR-130b-5p MIMAT0004680 | TTCGGACTCTTTCCCTGTTG | ACUCUUUCCCUGUUGCACUAC | GTGCAGGGTCCGAGGTCAGAGCCACCTGGGCAATTTTTTTTTTTGTAGTG |
| 15 | >hsa-miR-3675-5p MIMAT0018098 | TGGTATGGGGCTTCTGTAGA | UAUGGGGCUUCUGUAGAGAUUUC | GTGCAGGGTCCGAGGTCAGAGCCACCTGGGCAATTTTTTTTTTTGAAATC |
| 15 | >hsa-miR-3145-5p MIMAT0019205 | TTCGGAACTCCAAACACTCAA | AACUCCAAACACUCAAAACUCA | GTGCAGGGTCCGAGGTCAGAGCCACCTGGGCAATTTTTTTTTTTGAGTTT |
| 15 | >hsa-miR-589-5p MIMAT0004799 | TGGTGAGAACCACGTCTGC | UGAGAACCACGUCUGCUCUGAG | GTGCAGGGTCCGAGGTCAGAGCCACCTGGGCAATTTTTTTTTTTCTCAGA |
| 15 | >hsa-miR-7-1-3p MIMAT0004553 | TTCGGCAACAAATCACAGTCT | CAACAAAUCACAGUCUGCCAUA | GTGCAGGGTCCGAGGTCAGAGCCACCTGGGCAATTTTTTTTTTTATGGCA |
| 15 | >hsa-miR-455-5p MIMAT0003150 | TCGGTATGTGCCTTTGGACT | UAUGUGCCUUUGGACUACAUCG | GTGCAGGGTCCGAGGTCAGAGCCACCTGGGCAATTTTTTTTTTTCGATGT |
| 15 | >hsa-miR-224-5p MIMAT0000281 | TTCGGCAAGTCACTAGTGGT | CAAGUCACUAGUGGUUCCGUU | GTGCAGGGTCCGAGGTCAGAGCCACCTGGGCAATTTTTTTTTTTAACGGA |
| 15 | >hsa-miR-20a-5p MIMAT0000075 | TCGGTAAAGTGCTTATAGTGC | UAAAGUGCUUAUAGUGCAGGUAG | GTGCAGGGTCCGAGGTCAGAGCCACCTGGGCAATTTTTTTTTTTCTACCT |
| 16 | >hsa-miR-16-2-3p MIMAT0004518 | TTCGGCCAATATTACTGTGCT | CCAAUAUUACUGUGCUGCUUUA | GTGCAGGGTCCGAGGTCAGAGCCACCTGGGCAATTTTTTTTTTTAAAGCA |
| 16 | >hsa-miR-340-3p MIMAT0000750 | TCGGTCCGTCTCAGTTACTT | UCCGUCUCAGUUACUUUAUAGC | GTGCAGGGTCCGAGGTCAGAGCCACCTGGGCAATTTTTTTTTTTGCTATA |
| 16 | >hsa-miR-30c-5p MIMAT0000244 | TCGGTGTAAACATCCTACACT | UGUAAACAUCCUACACUCUCAGC | GTGCAGGGTCCGAGGTCAGAGCCACCTGGGCAATTTTTTTTTTTGCTGAG |
| 16 | >hsa-miR-544a MIMAT0003164 | GTCGGATTCTGCATTTTTAGC | AUUCUGCAUUUUUAGCAAGUUC | GTGCAGGGTCCGAGGTCAGAGCCACCTGGGCAATTTTTTTTTTTGAACTT |
| 16 | >hsa-miR-874-3p MIMAT0004911 | TCTGCCCTGGCCCGAGG | CUGCCCUGGCCCGAGGGACCGA | GTGCAGGGTCCGAGGTCAGAGCCACCTGGGCAATTTTTTTTTTTCGGTCC |
| 16 | >hsa-miR-125b-5p MIMAT0000423 | TGGTCCCTGAGACCCTAAC | UCCCUGAGACCCUAACUUGUGA | GTGCAGGGTCCGAGGTCAGAGCCACCTGGGCAATTTTTTTTTTTCACAAG |
| 16 | >hsa-miR-4306 MIMAT0016858 | TCGGTCGGTGGAGAGAAAG | UGGAGAGAAAGGCAGUA | GTGCAGGGTCCGAGGTCAGAGCCACCTGGGCAATTTTTTTTTTTACTGCC |
| 17 | >hsa-miR-542-3p MIMAT0003389 | TGTCGGTGTGACAGATTGATA | UGUGACAGAUUGAUAACUGAAA | GTGCAGGGTCCGAGGTCAGAGCCACCTGGGCAATTTTTTTTTTTCAGTTA |
| 17 | >hsa-miR-4770 MIMAT0019924 | CGGTCGGTGAGATGACACT | UGAGAUGACACUGUAGCU | GTGCAGGGTCCGAGGTCAGAGCCACCTGGGCAATTTTTTTTTTTAGCTAC |
| 17 | >hsa-miR-133b MIMAT0000770 | CGGTTTGGTCCCCTTCAAC | UUUGGUCCCCUUCAACCAGCUA | GTGCAGGGTCCGAGGTCAGAGCCACCTGGGCAATTTTTTTTTTTAGCTGG |
| 17 | >hsa-miR-223-3p MIMAT0000280 | GTCGGTGTCAGTTTGTCAAAT | UGUCAGUUUGUCAAAUACCCCA | GTGCAGGGTCCGAGGTCAGAGCCACCTGGGCAATTTTTTTTTTTGGGGTA |
| 17 | >hsa-miR-484 MIMAT0002174 | GGTCAGGCTCAGTCCCCT | UCAGGCUCAGUCCCCUCCCGAU | GTGCAGGGTCCGAGGTCAGAGCCACCTGGGCAATTTTTTTTTTTATCGGG |
| 17 | >hsa-miR-15a-5p MIMAT0000068 | TCGGTAGCAGCACATAATGG | UAGCAGCACAUAAUGGUUUGUG | GTGCAGGGTCCGAGGTCAGAGCCACCTGGGCAATTTTTTTTTTTCACAAA |
| 17 | >hsa-miR-490-3p MIMAT0002806 | GGCAACCTGGAGGACTCC | CAACCUGGAGGACUCCAUGCUG | GTGCAGGGTCCGAGGTCAGAGCCACCTGGGCAATTTTTTTTTTTCAGCAT |
| 18 | >hsa-miR-652-3p MIMAT0003322 | CGGAATGGCGCCACTAGG | AAUGGCGCCACUAGGGUUGUG | GTGCAGGGTCCGAGGTCAGAGCCACCTGGGCAATTTTTTTTTTTCACAAC |
| 18 | >hsa-miR-183-5p MIMAT0000261 | TCGGTATGGCACTGGTAGAA | UAUGGCACUGGUAGAAUUCACU | GTGCAGGGTCCGAGGTCAGAGCCACCTGGGCAATTTTTTTTTTTAGTGAA |
| 18 | >hsa-miR-210-3p MIMAT0000267 | GGCTGTGCGTGTGACAGC | CUGUGCGUGUGACAGCGGCUGA | GTGCAGGGTCCGAGGTCAGAGCCACCTGGGCAATTTTTTTTTTTCAGCCG |
| 18 | >hsa-miR-1260b MIMAT0015041 | GTCGGATCCCACCACTGC | AUCCCACCACUGCCACCAU | GTGCAGGGTCCGAGGTCAGAGCCACCTGGGCAATTTTTTTTTTTATGGTG |
| 18 | >hsa-miR-4538 MIMAT0019081 | TGGGAGCTTGGATGAGCTG | GAGCUUGGAUGAGCUGGGCUGA | GTGCAGGGTCCGAGGTCAGAGCCACCTGGGCAATTTTTTTTTTTCAGCCC |
| 18 | >hsa-miR-323b-3p MIMAT0015050 | TGGCCCAATACACGGTCGA | CCCAAUACACGGUCGACCUCUU | GTGCAGGGTCCGAGGTCAGAGCCACCTGGGCAATTTTTTTTTTTAAGAGG |
| 18 | >hsa-miR-550b-2-5p MIMAT0022737 | TGGATGTGCCTGAGGGAGT | AUGUGCCUGAGGGAGUAAGACA | GTGCAGGGTCCGAGGTCAGAGCCACCTGGGCAATTTTTTTTTTTGTCTTA |
| 19 | >hsa-miR-337-3p MIMAT0000754 | TCGGCTCCTATATGATGCCT | CUCCUAUAUGAUGCCUUUCUUC | GTGCAGGGTCCGAGGTCAGAGCCACCTGGGCAATTTTTTTTTTTGAAGAA |
| 19 | >hsa-miR-552 MIMAT0003215 | TTCGGAACAGGTGACTGGTT | AACAGGUGACUGGUUAGACAA | GTGCAGGGTCCGAGGTCAGAGCCACCTGGGCAATTTTTTTTTTTGTCTAA |
| 19 | >hsa-miR-1273c MIMAT0015017 | TGGGGCGACAAAACGAGAC | GGCGACAAAACGAGACCCUGUC | GTGCAGGGTCCGAGGTCAGAGCCACCTGGGCAATTTTTTTTTTTGACAGG |
| 19 | >hsa-miR-1244 MIMAT0005896 | TGAAGTAGTTGGTTTGTATGAG | AAGUAGUUGGUUUGUAUGAGAUGGUU | GTGCAGGGTCCGAGGTCAGAGCCACCTGGGCAATTTTTTTTTTTAACCAT |
| 19 | >hsa-miR-625-3p MIMAT0004808 | TTCGGGACTATAGAACTTTCC | GACUAUAGAACUUUCCCCCUCA | GTGCAGGGTCCGAGGTCAGAGCCACCTGGGCAATTTTTTTTTTTGAGGGG |
| 19 | >hsa-miR-376a-3p MIMAT0000729 | GGTCGGATCATAGAGGAAAAT | AUCAUAGAGGAAAAUCCACGU | GTGCAGGGTCCGAGGTCAGAGCCACCTGGGCAATTTTTTTTTTTACGTGG |
| 19 | >hsa-miR-142-5p MIMAT0000433 | TGTCGGCATAAAGTAGAAAGC | CAUAAAGUAGAAAGCACUACU | GTGCAGGGTCCGAGGTCAGAGCCACCTGGGCAATTTTTTTTTTTAGTAGT |
| 20 | >hsa-miR-523-5p MIMAT0005449 | GGCTCTAGAGGGAAGCGC | CUCUAGAGGGAAGCGCUUUCUG | GTGCAGGGTCCGAGGTCAGAGCCACCTGGGCAATTTTTTTTTTTCAGAAA |
| 20 | >hsa-miR-30a-3p MIMAT0000088 | TCGGCTTTCAGTCGGATGTT | CUUUCAGUCGGAUGUUUGCAGC | GTGCAGGGTCCGAGGTCAGAGCCACCTGGGCAATTTTTTTTTTTGCTGCA |
| 20 | >hsa-miR-574-3p MIMAT0003239 | TGGCACGCTCATGCACACA | CACGCUCAUGCACACACCCACA | GTGCAGGGTCCGAGGTCAGAGCCACCTGGGCAATTTTTTTTTTTGTGGGT |
| 20 | >hsa-miR-518e-3p MIMAT0002861 | TCGGAAAGCGCTTCCCTTC | AAAGCGCUUCCCUUCAGAGUG | GTGCAGGGTCCGAGGTCAGAGCCACCTGGGCAATTTTTTTTTTTCACTCT |
| 20 | >hsa-miR-1261 MIMAT0005913 | TGGTCGGATGGATAAGGCTT | AUGGAUAAGGCUUUGGCUU | GTGCAGGGTCCGAGGTCAGAGCCACCTGGGCAATTTTTTTTTTTAAGCCA |
| 20 | >hsa-miR-93-3p MIMAT0004509 | TGGACTGCTGAGCTAGCAC | ACUGCUGAGCUAGCACUUCCCG | GTGCAGGGTCCGAGGTCAGAGCCACCTGGGCAATTTTTTTTTTTCGGGAA |
| 20 | >hsa-miR-145-5p MIMAT0000437 | GGGTCCAGTTTTCCCAGGA | GUCCAGUUUUCCCAGGAAUCCCU | GTGCAGGGTCCGAGGTCAGAGCCACCTGGGCAATTTTTTTTTTTAGGGAT |
| 21 | >hsa-miR-33b-5p MIMAT0003301 | GTCGGGTGCATTGCTGTTG | GUGCAUUGCUGUUGCAUUGC | GTGCAGGGTCCGAGGTCAGAGCCACCTGGGCAATTTTTTTTTTTGCAATG |
| 21 | >hsa-miR-3121-5p MIMAT0019199 | TTCGGTCCTTTGCCTATTCTA | UCCUUUGCCUAUUCUAUUUAAG | GTGCAGGGTCCGAGGTCAGAGCCACCTGGGCAATTTTTTTTTTTCTTAAA |
| 21 | >hsa-miR-301a-3p MIMAT0000688 | TCGGCAGTGCAATAGTATTGT | CAGUGCAAUAGUAUUGUCAAAGC | GTGCAGGGTCCGAGGTCAGAGCCACCTGGGCAATTTTTTTTTTTGCTTTG |
| 21 | >hsa-miR-15b-5p MIMAT0000417 | CGGTAGCAGCACATCATGG | UAGCAGCACAUCAUGGUUUACA | GTGCAGGGTCCGAGGTCAGAGCCACCTGGGCAATTTTTTTTTTTGTAAAC |
| 21 | >hsa-miR-301b-3p MIMAT0004958 | TCGGCAGTGCAATGATATTGT | CAGUGCAAUGAUAUUGUCAAAGC | GTGCAGGGTCCGAGGTCAGAGCCACCTGGGCAATTTTTTTTTTTGCTTTG |
| 21 | >hsa-miR-3689b-3p MIMAT0018181 | CGGCTGGGAGGTGTGATAT | CUGGGAGGUGUGAUAUUGUGGU | GTGCAGGGTCCGAGGTCAGAGCCACCTGGGCAATTTTTTTTTTTACCACA |
| 21 | >hsa-miR-1262 MIMAT0005914 | TTCGGATGGGTGAATTTGTAG | AUGGGUGAAUUUGUAGAAGGAU | GTGCAGGGTCCGAGGTCAGAGCCACCTGGGCAATTTTTTTTTTTATCCTT |
| 22 | >hsa-miR-517a-3p MIMAT0002852 | TCGGATCGTGCATCCCTTTA | AUCGUGCAUCCCUUUAGAGUGU | GTGCAGGGTCCGAGGTCAGAGCCACCTGGGCAATTTTTTTTTTTACACTC |
| 22 | >hsa-miR-449b-5p MIMAT0003327 | TTCGGAGGCAGTGTATTGTTA | AGGCAGUGUAUUGUUAGCUGGC | GTGCAGGGTCCGAGGTCAGAGCCACCTGGGCAATTTTTTTTTTTGCCAGC |
| 22 | >hsa-miR-3135b MIMAT0018985 | TGGGCTGGAGCGAGTGCA | GGCUGGAGCGAGUGCAGUGGUG | GTGCAGGGTCCGAGGTCAGAGCCACCTGGGCAATTTTTTTTTTTCACCAC |
| 22 | >hsa-miR-378c MIMAT0016847 | GACTGGACTTGGAGTCAGAA | ACUGGACUUGGAGUCAGAAGAGUGG | GTGCAGGGTCCGAGGTCAGAGCCACCTGGGCAATTTTTTTTTTTCCACTC |
| 22 | >hsa-miR-525-5p MIMAT0002838 | CGGCTCCAGAGGGATGCA | CUCCAGAGGGAUGCACUUUCU | GTGCAGGGTCCGAGGTCAGAGCCACCTGGGCAATTTTTTTTTTTAGAAAG |
| 22 | >hsa-miR-92b-5p MIMAT0004792 | GAGGGACGGGACGCGGT | AGGGACGGGACGCGGUGCAGUG | GTGCAGGGTCCGAGGTCAGAGCCACCTGGGCAATTTTTTTTTTTCACTGC |
| 22 | >hsa-miR-33a-5p MIMAT0000091 | TTCGGGTGCATTGTAGTTGC | GUGCAUUGUAGUUGCAUUGCA | GTGCAGGGTCCGAGGTCAGAGCCACCTGGGCAATTTTTTTTTTTGCAATG |
| 23 | >hsa-miR-181c-3p MIMAT0004559 | CGGAACCATCGACCGTTGA | AACCAUCGACCGUUGAGUGGAC | GTGCAGGGTCCGAGGTCAGAGCCACCTGGGCAATTTTTTTTTTTGTCCAC |
| 23 | >hsa-miR-151a-5p MIMAT0004697 | CGGTCGAGGAGCTCACAG | UCGAGGAGCUCACAGUCUAGU | GTGCAGGGTCCGAGGTCAGAGCCACCTGGGCAATTTTTTTTTTTACTAGA |
| 23 | >hsa-miR-25-3p MIMAT0000081 | CGGCATTGCACTTGTCTCG | CAUUGCACUUGUCUCGGUCUGA | GTGCAGGGTCCGAGGTCAGAGCCACCTGGGCAATTTTTTTTTTTCAGACC |
| 23 | >hsa-let-7f-2-3p MIMAT0004487 | TTCGGCTATACAGTCTACTGT | CUAUACAGUCUACUGUCUUUCC | GTGCAGGGTCCGAGGTCAGAGCCACCTGGGCAATTTTTTTTTTTGGAAAG |
| 23 | >hsa-miR-19b-1-5p MIMAT0004491 | CGGAGTTTTGCAGGTTTGCA | AGUUUUGCAGGUUUGCAUCCAGC | GTGCAGGGTCCGAGGTCAGAGCCACCTGGGCAATTTTTTTTTTTGCTGGA |
| 23 | >hsa-miR-452-3p MIMAT0001636 | TCGGCTCATCTGCAAAGAAG | CUCAUCUGCAAAGAAGUAAGUG | GTGCAGGGTCCGAGGTCAGAGCCACCTGGGCAATTTTTTTTTTTCACTTA |
| 23 | >hsa-miR-29c-3p MIMAT0000681 | GTCGGTAGCACCATTTGAAAT | UAGCACCAUUUGAAAUCGGUUA | GTGCAGGGTCCGAGGTCAGAGCCACCTGGGCAATTTTTTTTTTTAACCGA |
| 24 | >hsa-miR-1321 MIMAT0005952 | GGTCGGCAGGGAGGTGAA | CAGGGAGGUGAAUGUGAU | GTGCAGGGTCCGAGGTCAGAGCCACCTGGGCAATTTTTTTTTTTATCACA |
| 24 | >hsa-miR-21-3p MIMAT0004494 | TCGGCAACACCAGTCGATG | CAACACCAGUCGAUGGGCUGU | GTGCAGGGTCCGAGGTCAGAGCCACCTGGGCAATTTTTTTTTTTACAGCC |
| 24 | >hsa-miR-3186-5p MIMAT0015067 | GGCAGGCGTCTGTCTACG | CAGGCGUCUGUCUACGUGGCUU | GTGCAGGGTCCGAGGTCAGAGCCACCTGGGCAATTTTTTTTTTTAAGCCA |
| 24 | >hsa-miR-30d-3p MIMAT0004551 | TTCGGCTTTCAGTCAGATGTT | CUUUCAGUCAGAUGUUUGCUGC | GTGCAGGGTCCGAGGTCAGAGCCACCTGGGCAATTTTTTTTTTTGCAGCA |
| 24 | >hsa-miR-3960 MIMAT0019337 | TGGGCGGCGGCGGAGG | GGCGGCGGCGGAGGCGGGGG | GTGCAGGGTCCGAGGTCAGAGCCACCTGGGCAATTTTTTTTTTTCCCCCG |
| 24 | >hsa-miR-3162-3p MIMAT0019213 | TGGTCCCTACCCCTCCAC | UCCCUACCCCUCCACUCCCCA | GTGCAGGGTCCGAGGTCAGAGCCACCTGGGCAATTTTTTTTTTTGGGGAG |
| 24 | >hsa-miR-3184-5p MIMAT0015064 | TGAGGGGCCTCAGACCGA | UGAGGGGCCUCAGACCGAGCUUUU | GTGCAGGGTCCGAGGTCAGAGCCACCTGGGCAATTTTTTTTTTTAAAAGC |
| 25 | >hsa-miR-548al MIMAT0019024 | TTCGGAACGGCAATGACTTTT | AACGGCAAUGACUUUUGUACCA | GTGCAGGGTCCGAGGTCAGAGCCACCTGGGCAATTTTTTTTTTTGGTACA |
| 25 | >hsa-miR-515-5p MIMAT0002826 | TGGTTCTCCAAAAGAAAGCAC | UUCUCCAAAAGAAAGCACUUUCUG | GTGCAGGGTCCGAGGTCAGAGCCACCTGGGCAATTTTTTTTTTTCAGAAA |
| 25 | >hsa-miR-423-3p MIMAT0001340 | TAGCTCGGTCTGAGGCCC | AGCUCGGUCUGAGGCCCCUCAGU | GTGCAGGGTCCGAGGTCAGAGCCACCTGGGCAATTTTTTTTTTTACTGAG |
| 25 | >hsa-miR-3124-3p MIMAT0019200 | TGGACTTTCCTCACTCCCG | ACUUUCCUCACUCCCGUGAAGU | GTGCAGGGTCCGAGGTCAGAGCCACCTGGGCAATTTTTTTTTTTACTTCA |
| 25 | >hsa-miR-1247-5p MIMAT0005899 | TGACCCGTCCCGTTCGTC | ACCCGUCCCGUUCGUCCCCGGA | GTGCAGGGTCCGAGGTCAGAGCCACCTGGGCAATTTTTTTTTTTCCGGGG |
| 25 | >hsa-miR-10a-5p MIMAT0000253 | TGGTACCCTGTAGATCCGAA | UACCCUGUAGAUCCGAAUUUGUG | GTGCAGGGTCCGAGGTCAGAGCCACCTGGGCAATTTTTTTTTTTCACAAA |
| 25 | >hsa-miR-19b-3p MIMAT0000074 | CGGTGTGCAAATCCATGCAA | UGUGCAAAUCCAUGCAAAACUGA | GTGCAGGGTCCGAGGTCAGAGCCACCTGGGCAATTTTTTTTTTTCAGTTT |
| 26 | >hsa-miR-4296 MIMAT0016845 | CGGTCGGATGTGGGCTCA | AUGUGGGCUCAGGCUCA | GTGCAGGGTCCGAGGTCAGAGCCACCTGGGCAATTTTTTTTTTTGAGCCT |
| 26 | >hsa-miR-548a-3p MIMAT0003251 | TTCGGCAAAACTGGCAATTAC | CAAAACUGGCAAUUACUUUUGC | GTGCAGGGTCCGAGGTCAGAGCCACCTGGGCAATTTTTTTTTTTGCAAAA |
| 26 | >hsa-miR-337-5p MIMAT0004695 | TTCGGGAACGGCTTCATACA | GAACGGCUUCAUACAGGAGUU | GTGCAGGGTCCGAGGTCAGAGCCACCTGGGCAATTTTTTTTTTTAACTCC |
| 26 | >hsa-let-7b-5p MIMAT0000063 | TCGGTGAGGTAGTAGGTTGT | UGAGGUAGUAGGUUGUGUGGUU | GTGCAGGGTCCGAGGTCAGAGCCACCTGGGCAATTTTTTTTTTTAACCAC |
| 26 | >hsa-miR-342-5p MIMAT0004694 | TCGGAGGGGTGCTATCTGT | AGGGGUGCUAUCUGUGAUUGA | GTGCAGGGTCCGAGGTCAGAGCCACCTGGGCAATTTTTTTTTTTCAATCA |
| 26 | >hsa-miR-2861 MIMAT0013802 | CGGGGGGCCTGGCGGT | GGGGCCUGGCGGUGGGCGG | GTGCAGGGTCCGAGGTCAGAGCCACCTGGGCAATTTTTTTTTTTCCGCCC |
| 26 | >hsa-miR-758-5p MIMAT0022929 | CGGGATGGTTGACCAGAGA | GAUGGUUGACCAGAGAGCACAC | GTGCAGGGTCCGAGGTCAGAGCCACCTGGGCAATTTTTTTTTTTGTGTGC |
| 27 | >hsa-miR-1587 MIMAT0019077 | CGGTTGGGCTGGGCTGG | UUGGGCUGGGCUGGGUUGGG | GTGCAGGGTCCGAGGTCAGAGCCACCTGGGCAATTTTTTTTTTTCCCAAC |
| 27 | >hsa-miR-103a-2-5p MIMAT0009196 | CGGAGCTTCTTTACAGTGCT | AGCUUCUUUACAGUGCUGCCUUG | GTGCAGGGTCCGAGGTCAGAGCCACCTGGGCAATTTTTTTTTTTCAAGGC |
| 27 | >hsa-miR-323a-3p MIMAT0000755 | TCGGCACATTACACGGTCG | CACAUUACACGGUCGACCUCU | GTGCAGGGTCCGAGGTCAGAGCCACCTGGGCAATTTTTTTTTTTAGAGGT |
| 27 | >hsa-miR-365b-3p MIMAT0022834 | GTCGGTAATGCCCCTAAAAAT | UAAUGCCCCUAAAAAUCCUUAU | GTGCAGGGTCCGAGGTCAGAGCCACCTGGGCAATTTTTTTTTTTATAAGG |
| 27 | >hsa-miR-181a-2-3p MIMAT0004558 | TGGACCACTGACCGTTGAC | ACCACUGACCGUUGACUGUACC | GTGCAGGGTCCGAGGTCAGAGCCACCTGGGCAATTTTTTTTTTTGGTACA |
| 27 | >hsa-miR-1183 MIMAT0005828 | CACTGTAGGTGATGGTGAGAG | CACUGUAGGUGAUGGUGAGAGUGGGCA | GTGCAGGGTCCGAGGTCAGAGCCACCTGGGCAATTTTTTTTTTTGCCCAC |
| 27 | >hsa-miR-517b-3p MIMAT0002857 | TCGGATCGTGCATCCCTTTA | AUCGUGCAUCCCUUUAGAGUGU | GTGCAGGGTCCGAGGTCAGAGCCACCTGGGCAATTTTTTTTTTTACACTC |
| 28 | >hsa-miR-144-5p MIMAT0004600 | GTCGGGGATATCATCATATAC | GGAUAUCAUCAUAUACUGUAAG | GTGCAGGGTCCGAGGTCAGAGCCACCTGGGCAATTTTTTTTTTTCTTACA |
| 28 | >hsa-miR-3129-3p MIMAT0019202 | GTCGGAAACTAATCTCTACAC | AAACUAAUCUCUACACUGCUGC | GTGCAGGGTCCGAGGTCAGAGCCACCTGGGCAATTTTTTTTTTTGCAGCA |
| 28 | >hsa-miR-1238 MIMAT0005593 | TTCGGCTTCCTCGTCTGTC | CUUCCUCGUCUGUCUGCCCC | GTGCAGGGTCCGAGGTCAGAGCCACCTGGGCAATTTTTTTTTTTGGGGCA |
| 28 | >hsa-let-7e-3p MIMAT0004485 | TGGCTATACGGCCTCCTAG | CUAUACGGCCUCCUAGCUUUCC | GTGCAGGGTCCGAGGTCAGAGCCACCTGGGCAATTTTTTTTTTTGGAAAG |
| 28 | >hsa-miR-625-5p MIMAT0003294 | TTCGGAGGGGGAAAGTTCTA | AGGGGGAAAGUUCUAUAGUCC | GTGCAGGGTCCGAGGTCAGAGCCACCTGGGCAATTTTTTTTTTTGGACTA |
| 28 | >hsa-miR-18b-5p MIMAT0001412 | TGGTAAGGTGCATCTAGTGC | UAAGGUGCAUCUAGUGCAGUUAG | GTGCAGGGTCCGAGGTCAGAGCCACCTGGGCAATTTTTTTTTTTCTAACT |
| 28 | >hsa-miR-1246 MIMAT0005898 | CGGTCGGAATGGATTTTTGG | AAUGGAUUUUUGGAGCAGG | GTGCAGGGTCCGAGGTCAGAGCCACCTGGGCAATTTTTTTTTTTCCTGCT |
| 29 | >hsa-miR-193a-3p MIMAT0000459 | TCGGAACTGGCCTACAAAGT | AACUGGCCUACAAAGUCCCAGU | GTGCAGGGTCCGAGGTCAGAGCCACCTGGGCAATTTTTTTTTTTACTGGG |
| 29 | >hsa-miR-3657 MIMAT0018077 | GTCGGTGTGTCCCATTATTG | UGUGUCCCAUUAUUGGUGAUU | GTGCAGGGTCCGAGGTCAGAGCCACCTGGGCAATTTTTTTTTTTAATCAC |
| 29 | >hsa-miR-424-5p MIMAT0001341 | TCGGCAGCAGCAATTCATGT | CAGCAGCAAUUCAUGUUUUGAA | GTGCAGGGTCCGAGGTCAGAGCCACCTGGGCAATTTTTTTTTTTCAAAAC |
| 29 | >hsa-miR-636 MIMAT0003306 | GTGTGCTTGCTCGTCCCG | UGUGCUUGCUCGUCCCGCCCGCA | GTGCAGGGTCCGAGGTCAGAGCCACCTGGGCAATTTTTTTTTTTGCGGGC |
| 29 | >hsa-miR-130a-3p MIMAT0000425 | GTCGGCAGTGCAATGTTAAAA | CAGUGCAAUGUUAAAAGGGCAU | GTGCAGGGTCCGAGGTCAGAGCCACCTGGGCAATTTTTTTTTTTATGCCC |
| 29 | >hsa-miR-378i MIMAT0019074 | TCGGACTGGACTAGGAGTC | ACUGGACUAGGAGUCAGAAGG | GTGCAGGGTCCGAGGTCAGAGCCACCTGGGCAATTTTTTTTTTTCCTTCT |
| 29 | >hsa-miR-16-5p MIMAT0000069 | TTCGGTAGCAGCACGTAAATA | UAGCAGCACGUAAAUAUUGGCG | GTGCAGGGTCCGAGGTCAGAGCCACCTGGGCAATTTTTTTTTTTCGCCAA |
| 30 | >hsa-miR-22-3p MIMAT0000077 | CGGAAGCTGCCAGTTGAAG | AAGCUGCCAGUUGAAGAACUGU | GTGCAGGGTCCGAGGTCAGAGCCACCTGGGCAATTTTTTTTTTTACAGTT |
| 30 | >hsa-miR-10b-5p MIMAT0000254 | TGGTACCCTGTAGAACCGAA | UACCCUGUAGAACCGAAUUUGUG | GTGCAGGGTCCGAGGTCAGAGCCACCTGGGCAATTTTTTTTTTTCACAAA |
| 30 | >hsa-miR-193a-5p MIMAT0004614 | TGTGGGTCTTTGCGGGCG | UGGGUCUUUGCGGGCGAGAUGA | GTGCAGGGTCCGAGGTCAGAGCCACCTGGGCAATTTTTTTTTTTCATCTC |
| 30 | >hsa-miR-21-5p MIMAT0000076 | TTCGGTAGCTTATCAGACTGA | UAGCUUAUCAGACUGAUGUUGA | GTGCAGGGTCCGAGGTCAGAGCCACCTGGGCAATTTTTTTTTTTCAACAT |
| 30 | >hsa-miR-3178 MIMAT0015055 | TTCGGGGGGCGCGGCC | GGGGCGCGGCCGGAUCG | GTGCAGGGTCCGAGGTCAGAGCCACCTGGGCAATTTTTTTTTTTCGATCC |
| 30 | >hsa-miR-4687-5p MIMAT0019774 | TGCAGCCCTCCTCCCGC | CAGCCCUCCUCCCGCACCCAAA | GTGCAGGGTCCGAGGTCAGAGCCACCTGGGCAATTTTTTTTTTTGGGTGC |
| 30 | >hsa-miR-194-5p MIMAT0000460 | TCGGTGTAACAGCAACTCCA | UGUAACAGCAACUCCAUGUGGA | GTGCAGGGTCCGAGGTCAGAGCCACCTGGGCAATTTTTTTTTTTCCACAT |
| 31 | >hsa-miR-200c-3p MIMAT0000617 | CGGTAATACTGCCGGGTAAT | UAAUACUGCCGGGUAAUGAUGGA | GTGCAGGGTCCGAGGTCAGAGCCACCTGGGCAATTTTTTTTTTTCCATCA |
| 31 | >hsa-miR-374b-5p MIMAT0004955 | GTCGGATATAATACAACCTGC | AUAUAAUACAACCUGCUAAGUG | GTGCAGGGTCCGAGGTCAGAGCCACCTGGGCAATTTTTTTTTTTCACTTA |
| 31 | >hsa-miR-514b-5p MIMAT0015087 | TGGTTCTCAAGAGGGAGGC | UUCUCAAGAGGGAGGCAAUCAU | GTGCAGGGTCCGAGGTCAGAGCCACCTGGGCAATTTTTTTTTTTATGATT |
| 31 | >hsa-miR-576-5p MIMAT0003241 | GTCGGATTCTAATTTCTCCAC | AUUCUAAUUUCUCCACGUCUUU | GTGCAGGGTCCGAGGTCAGAGCCACCTGGGCAATTTTTTTTTTTAAAGAC |
| 31 | >hsa-miR-217 MIMAT0000274 | TGGTACTGCATCAGGAACTG | UACUGCAUCAGGAACUGAUUGGA | GTGCAGGGTCCGAGGTCAGAGCCACCTGGGCAATTTTTTTTTTTCCAATC |
| 31 | >hsa-miR-107 MIMAT0000104 | GGAGCAGCATTGTACAGGG | AGCAGCAUUGUACAGGGCUAUCA | GTGCAGGGTCCGAGGTCAGAGCCACCTGGGCAATTTTTTTTTTTGATAGC |
| 31 | >hsa-let-7d-5p MIMAT0000065 | CGGAGAGGTAGTAGGTTGC | AGAGGUAGUAGGUUGCAUAGUU | GTGCAGGGTCCGAGGTCAGAGCCACCTGGGCAATTTTTTTTTTTAACTAT |
| 32 | >hsa-miR-1236 MIMAT0005591 | TGGCCTCTTCCCCTTGTCT | CCUCUUCCCCUUGUCUCUCCAG | GTGCAGGGTCCGAGGTCAGAGCCACCTGGGCAATTTTTTTTTTTCTGGAG |
| 32 | >hsa-miR-374a-5p MIMAT0000727 | TGTCGGTTATAATACAACCTGA | UUAUAAUACAACCUGAUAAGUG | GTGCAGGGTCCGAGGTCAGAGCCACCTGGGCAATTTTTTTTTTTCACTTA |
| 32 | >hsa-miR-3200-3p MIMAT0015085 | TGGCACCTTGCGCTACTCA | CACCUUGCGCUACUCAGGUCUG | GTGCAGGGTCCGAGGTCAGAGCCACCTGGGCAATTTTTTTTTTTCAGACC |
| 32 | >hsa-miR-1283 MIMAT0005799 | TCGGTCTACAAAGGAAAGCG | UCUACAAAGGAAAGCGCUUUCU | GTGCAGGGTCCGAGGTCAGAGCCACCTGGGCAATTTTTTTTTTTAGAAAG |
| 32 | >hsa-miR-100-5p MIMAT0000098 | CGGAACCCGTAGATCCGAA | AACCCGUAGAUCCGAACUUGUG | GTGCAGGGTCCGAGGTCAGAGCCACCTGGGCAATTTTTTTTTTTCACAAG |
| 32 | >hsa-miR-148b-3p MIMAT0000759 | TCGGTCAGTGCATCACAGAA | UCAGUGCAUCACAGAACUUUGU | GTGCAGGGTCCGAGGTCAGAGCCACCTGGGCAATTTTTTTTTTTACAAAG |
| 32 | >hsa-miR-378e MIMAT0018927 | TGTCGGACTGGACTTGGAG | ACUGGACUUGGAGUCAGGA | GTGCAGGGTCCGAGGTCAGAGCCACCTGGGCAATTTTTTTTTTTCCTGAC |
| 33 | >hsa-miR-196a-3p MIMAT0004562 | CGGCGGCAACAAGAAACTG | CGGCAACAAGAAACUGCCUGAG | GTGCAGGGTCCGAGGTCAGAGCCACCTGGGCAATTTTTTTTTTTCTCAGG |
| 33 | >hsa-miR-205-5p MIMAT0000266 | TGGTCCTTCATTCCACCGG | UCCUUCAUUCCACCGGAGUCUG | GTGCAGGGTCCGAGGTCAGAGCCACCTGGGCAATTTTTTTTTTTCAGACT |
| 33 | >hsa-miR-139-5p MIMAT0000250 | GGTCTACAGTGCACGTGTC | UCUACAGUGCACGUGUCUCCAGU | GTGCAGGGTCCGAGGTCAGAGCCACCTGGGCAATTTTTTTTTTTACTGGA |
| 33 | >hsa-miR-191-3p MIMAT0001618 | TGGGCTGCGCTTGGATTTC | GCUGCGCUUGGAUUUCGUCCCC | GTGCAGGGTCCGAGGTCAGAGCCACCTGGGCAATTTTTTTTTTTGGGGAC |
| 33 | >hsa-miR-378d MIMAT0018926 | TGTCGGACTGGACTTGGAG | ACUGGACUUGGAGUCAGAAA | GTGCAGGGTCCGAGGTCAGAGCCACCTGGGCAATTTTTTTTTTTCTGACT |
| 33 | >hsa-miR-590-5p MIMAT0003258 | TGTCGGGAGCTTATTCATAAAA | GAGCUUAUUCAUAAAAGUGCAG | GTGCAGGGTCCGAGGTCAGAGCCACCTGGGCAATTTTTTTTTTTCTGCAC |
| 33 | >hsa-miR-373-3p MIMAT0000726 | CGGGAAGTGCTTCGATTTTG | GAAGUGCUUCGAUUUUGGGGUGU | GTGCAGGGTCCGAGGTCAGAGCCACCTGGGCAATTTTTTTTTTTACACCC |
| 34 | >hsa-miR-485-3p MIMAT0002176 | TGGGTCATACACGGCTCTC | GUCAUACACGGCUCUCCUCUCU | GTGCAGGGTCCGAGGTCAGAGCCACCTGGGCAATTTTTTTTTTTAGAGAG |
| 34 | >hsa-miR-190b MIMAT0004929 | TGGTCGGTGATATGTTTGATAT | UGAUAUGUUUGAUAUUGGGUU | GTGCAGGGTCCGAGGTCAGAGCCACCTGGGCAATTTTTTTTTTTAACCCA |
| 34 | >hsa-miR-124-3p MIMAT0000422 | TCGGTAAGGCACGCGGTG | UAAGGCACGCGGUGAAUGCC | GTGCAGGGTCCGAGGTCAGAGCCACCTGGGCAATTTTTTTTTTTGGCATT |
| 34 | >hsa-miR-574-5p MIMAT0004795 | TGGTGAGTGTGTGTGTGTGA | UGAGUGUGUGUGUGUGAGUGUGU | GTGCAGGGTCCGAGGTCAGAGCCACCTGGGCAATTTTTTTTTTTACACAC |
| 34 | >hsa-miR-99a-3p MIMAT0004511 | CGGCAAGCTCGCTTCTATG | CAAGCUCGCUUCUAUGGGUCUG | GTGCAGGGTCCGAGGTCAGAGCCACCTGGGCAATTTTTTTTTTTCAGACC |
| 34 | >hsa-miR-3150a-5p MIMAT0019206 | TGGCAACCTCGACGATCTC | CAACCUCGACGAUCUCCUCAGC | GTGCAGGGTCCGAGGTCAGAGCCACCTGGGCAATTTTTTTTTTTGCTGAG |
| 34 | >hsa-miR-517c-3p MIMAT0002866 | TTCGGATCGTGCATCCTTTTA | AUCGUGCAUCCUUUUAGAGUGU | GTGCAGGGTCCGAGGTCAGAGCCACCTGGGCAATTTTTTTTTTTACACTC |
| 35 | >hsa-miR-3911 MIMAT0018185 | TGGTGTGTGGATCCTGGAG | UGUGUGGAUCCUGGAGGAGGCA | GTGCAGGGTCCGAGGTCAGAGCCACCTGGGCAATTTTTTTTTTTGCCTCC |
| 35 | >hsa-miR-3198 MIMAT0015083 | GGGTGGAGTCCTGGGGAA | GUGGAGUCCUGGGGAAUGGAGA | GTGCAGGGTCCGAGGTCAGAGCCACCTGGGCAATTTTTTTTTTTCTCCAT |
| 35 | >hsa-miR-150-3p MIMAT0004610 | TGCTGGTACAGGCCTGGG | CUGGUACAGGCCUGGGGGACAG | GTGCAGGGTCCGAGGTCAGAGCCACCTGGGCAATTTTTTTTTTTCTGTCC |
| 35 | >hsa-miR-24-3p MIMAT0000080 | TGGTGGCTCAGTTCAGCAG | UGGCUCAGUUCAGCAGGAACAG | GTGCAGGGTCCGAGGTCAGAGCCACCTGGGCAATTTTTTTTTTTCTGTTC |
| 35 | >hsa-miR-429 MIMAT0001536 | GTCGGTAATACTGTCTGGTAA | UAAUACUGUCUGGUAAAACCGU | GTGCAGGGTCCGAGGTCAGAGCCACCTGGGCAATTTTTTTTTTTACGGTT |
| 35 | >hsa-miR-219a-5p MIMAT0000276 | TTCGGTGATTGTCCAAACGC | UGAUUGUCCAAACGCAAUUCU | GTGCAGGGTCCGAGGTCAGAGCCACCTGGGCAATTTTTTTTTTTAGAATT |
| 35 | >hsa-miR-377-3p MIMAT0000730 | TCGGATCACACAAAGGCAAC | AUCACACAAAGGCAACUUUUGU | GTGCAGGGTCCGAGGTCAGAGCCACCTGGGCAATTTTTTTTTTTACAAAA |
| 36 | >hsa-miR-339-5p MIMAT0000764 | GTCCCTGTCCTCCAGGAG | UCCCUGUCCUCCAGGAGCUCACG | GTGCAGGGTCCGAGGTCAGAGCCACCTGGGCAATTTTTTTTTTTCGTGAG |
| 36 | >hsa-miR-3127-5p MIMAT0014990 | TGGATCAGGGCTTGTGGAAT | AUCAGGGCUUGUGGAAUGGGAAG | GTGCAGGGTCCGAGGTCAGAGCCACCTGGGCAATTTTTTTTTTTCTTCCC |
| 36 | >hsa-miR-24-1-5p MIMAT0000079 | CGGTGCCTACTGAGCTGAT | UGCCUACUGAGCUGAUAUCAGU | GTGCAGGGTCCGAGGTCAGAGCCACCTGGGCAATTTTTTTTTTTACTGAT |
| 36 | >hsa-miR-195-5p MIMAT0000461 | GTCGGTAGCAGCACAGAAAT | UAGCAGCACAGAAAUAUUGGC | GTGCAGGGTCCGAGGTCAGAGCCACCTGGGCAATTTTTTTTTTTGCCAAT |
| 36 | >hsa-miR-3922-3p MIMAT0018197 | CGGTCTGGCCTTGACTTGA | UCUGGCCUUGACUUGACUCUUU | GTGCAGGGTCCGAGGTCAGAGCCACCTGGGCAATTTTTTTTTTTAAAGAG |
| 36 | >hsa-miR-374c-5p MIMAT0018443 | GTCGGATAATACAACCTGCTA | AUAAUACAACCUGCUAAGUGCU | GTGCAGGGTCCGAGGTCAGAGCCACCTGGGCAATTTTTTTTTTTAGCACT |
| 36 | >hsa-miR-582-5p MIMAT0003247 | TCGGTTACAGTTGTTCAACCA | UUACAGUUGUUCAACCAGUUACU | GTGCAGGGTCCGAGGTCAGAGCCACCTGGGCAATTTTTTTTTTTAGTAAC |
| 37 | >hsa-miR-103a-3p MIMAT0000101 | GGAGCAGCATTGTACAGGG | AGCAGCAUUGUACAGGGCUAUGA | GTGCAGGGTCCGAGGTCAGAGCCACCTGGGCAATTTTTTTTTTTCATAGC |
| 37 | >hsa-miR-526b-5p MIMAT0002835 | GGCTCTTGAGGGAAGCACT | CUCUUGAGGGAAGCACUUUCUGU | GTGCAGGGTCCGAGGTCAGAGCCACCTGGGCAATTTTTTTTTTTACAGAA |
| 37 | >hsa-miR-370-3p MIMAT0000722 | TGGCCTGCTGGGGTGGAA | GCCUGCUGGGGUGGAACCUGGU | GTGCAGGGTCCGAGGTCAGAGCCACCTGGGCAATTTTTTTTTTTACCAGG |
| 37 | >hsa-miR-3117-5p MIMAT0019197 | TGTCGGAGACACTATACGAG | AGACACUAUACGAGUCAUAU | GTGCAGGGTCCGAGGTCAGAGCCACCTGGGCAATTTTTTTTTTTATATGA |
| 37 | >hsa-miR-186-3p MIMAT0004612 | TCGGGCCCAAAGGTGAATTT | GCCCAAAGGUGAAUUUUUUGGG | GTGCAGGGTCCGAGGTCAGAGCCACCTGGGCAATTTTTTTTTTTCCCAAA |
| 37 | >hsa-miR-299-5p MIMAT0002890 | TGGTGGTTTACCGTCCCAC | UGGUUUACCGUCCCACAUACAU | GTGCAGGGTCCGAGGTCAGAGCCACCTGGGCAATTTTTTTTTTTATGTAT |
| 37 | >hsa-miR-548k MIMAT0005882 | TTCGGAAAAGTACTTGCGGAT | AAAAGUACUUGCGGAUUUUGCU | GTGCAGGGTCCGAGGTCAGAGCCACCTGGGCAATTTTTTTTTTTAGCAAA |
| 38 | >hsa-miR-96-5p MIMAT0000095 | CGGTTTGGCACTAGCACATT | UUUGGCACUAGCACAUUUUUGCU | GTGCAGGGTCCGAGGTCAGAGCCACCTGGGCAATTTTTTTTTTTAGCAAA |
| 38 | >hsa-miR-136-3p MIMAT0004606 | TTCGGCATCATCGTCTCAAAT | CAUCAUCGUCUCAAAUGAGUCU | GTGCAGGGTCCGAGGTCAGAGCCACCTGGGCAATTTTTTTTTTTAGACTC |
| 38 | >hsa-miR-20a-3p MIMAT0004493 | TCGGACTGCATTATGAGCAC | ACUGCAUUAUGAGCACUUAAAG | GTGCAGGGTCCGAGGTCAGAGCCACCTGGGCAATTTTTTTTTTTCTTTAA |
| 38 | >hsa-miR-203a-3p MIMAT0000264 | TTCGGGTGAAATGTTTAGGAC | GUGAAAUGUUUAGGACCACUAG | GTGCAGGGTCCGAGGTCAGAGCCACCTGGGCAATTTTTTTTTTTCTAGTG |
| 38 | >hsa-miR-30d-5p MIMAT0000245 | CGGTGTAAACATCCCCGAC | UGUAAACAUCCCCGACUGGAAG | GTGCAGGGTCCGAGGTCAGAGCCACCTGGGCAATTTTTTTTTTTCTTCCA |
| 38 | >hsa-miR-450a-5p MIMAT0001545 | TCGGTTTTGCGATGTGTTCC | UUUUGCGAUGUGUUCCUAAUAU | GTGCAGGGTCCGAGGTCAGAGCCACCTGGGCAATTTTTTTTTTTATATTA |
| 38 | >hsa-miR-548ap-3p MIMAT0021038 | TCGGTCGGAAAAACCACAATT | AAAAACCACAAUUACUUUU | GTGCAGGGTCCGAGGTCAGAGCCACCTGGGCAATTTTTTTTTTTAAAAGT |
| 39 | >hsa-miR-362-3p MIMAT0004683 | TTCGGAACACACCTATTCAAG | AACACACCUAUUCAAGGAUUCA | GTGCAGGGTCCGAGGTCAGAGCCACCTGGGCAATTTTTTTTTTTGAATCC |
| 39 | >hsa-miR-548w MIMAT0015060 | TCGGAAAAGTAACTGCGGTTT | AAAAGUAACUGCGGUUUUUGCCU | GTGCAGGGTCCGAGGTCAGAGCCACCTGGGCAATTTTTTTTTTTAGGCAA |
| 39 | >hsa-miR-199a-3p MIMAT0000232 | TCGGACAGTAGTCTGCACAT | ACAGUAGUCUGCACAUUGGUUA | GTGCAGGGTCCGAGGTCAGAGCCACCTGGGCAATTTTTTTTTTTAACCAA |
| 39 | >hsa-miR-27a-3p MIMAT0000084 | TTCGGTTCACAGTGGCTAAG | UUCACAGUGGCUAAGUUCCGC | GTGCAGGGTCCGAGGTCAGAGCCACCTGGGCAATTTTTTTTTTTGCGGAA |
| 39 | >hsa-miR-140-5p MIMAT0000431 | TCGGCAGTGGTTTTACCCTA | CAGUGGUUUUACCCUAUGGUAG | GTGCAGGGTCCGAGGTCAGAGCCACCTGGGCAATTTTTTTTTTTCTACCA |
| 39 | >hsa-miR-501-3p MIMAT0004774 | GGAATGCACCCGGGCAAG | AAUGCACCCGGGCAAGGAUUCU | GTGCAGGGTCCGAGGTCAGAGCCACCTGGGCAATTTTTTTTTTTAGAATC |
| 39 | >hsa-miR-3914 MIMAT0018188 | TTCGGAAGGAACCAGAAAATG | AAGGAACCAGAAAAUGAGAAGU | GTGCAGGGTCCGAGGTCAGAGCCACCTGGGCAATTTTTTTTTTTACTTCT |
| 40 | >hsa-miR-421 MIMAT0003339 | GTCGGATCAACAGACATTAATT | AUCAACAGACAUUAAUUGGGCGC | GTGCAGGGTCCGAGGTCAGAGCCACCTGGGCAATTTTTTTTTTTGCGCCC |
| 40 | >hsa-miR-3194-3p MIMAT0019218 | TGGAGCTCTGCTGCTCACT | AGCUCUGCUGCUCACUGGCAGU | GTGCAGGGTCCGAGGTCAGAGCCACCTGGGCAATTTTTTTTTTTACTGCC |
| 40 | >hsa-miR-425-3p MIMAT0001343 | CGGATCGGGAATGTCGTGT | AUCGGGAAUGUCGUGUCCGCCC | GTGCAGGGTCCGAGGTCAGAGCCACCTGGGCAATTTTTTTTTTTGGGCGG |
| 40 | >hsa-miR-154-3p MIMAT0000453 | TTCGGAATCATACACGGTTGA | AAUCAUACACGGUUGACCUAUU | GTGCAGGGTCCGAGGTCAGAGCCACCTGGGCAATTTTTTTTTTTAATAGG |
| 40 | >hsa-miR-3135a MIMAT0015001 | TGGTGCCTAGGCTGAGACT | UGCCUAGGCUGAGACUGCAGUG | GTGCAGGGTCCGAGGTCAGAGCCACCTGGGCAATTTTTTTTTTTCACTGC |
| 40 | >hsa-miR-542-5p MIMAT0003340 | GGTCGGGGATCATCATGTC | UCGGGGAUCAUCAUGUCACGAGA | GTGCAGGGTCCGAGGTCAGAGCCACCTGGGCAATTTTTTTTTTTCTCGTG |
| 40 | >hsa-miR-23a-5p MIMAT0004496 | TGGGGGTTCCTGGGGATG | GGGGUUCCUGGGGAUGGGAUUU | GTGCAGGGTCCGAGGTCAGAGCCACCTGGGCAATTTTTTTTTTTAAATCC |
| 41 | >hsa-miR-151a-3p MIMAT0000757 | TCGGCTAGACTGAAGCTCC | CUAGACUGAAGCUCCUUGAGG | GTGCAGGGTCCGAGGTCAGAGCCACCTGGGCAATTTTTTTTTTTCCTCAA |
| 41 | >hsa-miR-1256 MIMAT0005907 | TCGGAGGCATTGACTTCTCA | AGGCAUUGACUUCUCACUAGCU | GTGCAGGGTCCGAGGTCAGAGCCACCTGGGCAATTTTTTTTTTTAGCTAG |
| 41 | >hsa-miR-3621 MIMAT0018002 | GGCGCGGGTCGGGGTC | CGCGGGUCGGGGUCUGCAGG | GTGCAGGGTCCGAGGTCAGAGCCACCTGGGCAATTTTTTTTTTTCCTGCA |
| 41 | >hsa-miR-492 MIMAT0002812 | GAGGACCTGCGGGACAAG | AGGACCUGCGGGACAAGAUUCUU | GTGCAGGGTCCGAGGTCAGAGCCACCTGGGCAATTTTTTTTTTTAAGAAT |
| 41 | >hsa-miR-140-3p MIMAT0004597 | TTCGGTACCACAGGGTAGAA | UACCACAGGGUAGAACCACGG | GTGCAGGGTCCGAGGTCAGAGCCACCTGGGCAATTTTTTTTTTTCCGTGG |
| 41 | >hsa-miR-629-3p MIMAT0003298 | CGGGTTCTCCCAACGTAAG | GUUCUCCCAACGUAAGCCCAGC | GTGCAGGGTCCGAGGTCAGAGCCACCTGGGCAATTTTTTTTTTTGCTGGG |
| 41 | >hsa-miR-518a-5p MIMAT0005457 | TTCGGCTGCAAAGGGAAGC | CUGCAAAGGGAAGCCCUUUC | GTGCAGGGTCCGAGGTCAGAGCCACCTGGGCAATTTTTTTTTTTGAAAGG |
| 42 | >hsa-miR-320c MIMAT0005793 | TGTCGGAAAAGCTGGGTTGA | AAAAGCUGGGUUGAGAGGGU | GTGCAGGGTCCGAGGTCAGAGCCACCTGGGCAATTTTTTTTTTTACCCTC |
| 42 | >hsa-miR-188-5p MIMAT0000457 | TCGGCATCCCTTGCATGGT | CAUCCCUUGCAUGGUGGAGGG | GTGCAGGGTCCGAGGTCAGAGCCACCTGGGCAATTTTTTTTTTTCCCTCC |
| 42 | >hsa-miR-422a MIMAT0001339 | CGGACTGGACTTAGGGTCA | ACUGGACUUAGGGUCAGAAGGC | GTGCAGGGTCCGAGGTCAGAGCCACCTGGGCAATTTTTTTTTTTGCCTTC |
| 42 | >hsa-miR-769-5p MIMAT0003886 | TGGTGAGACCTCTGGGTTC | UGAGACCUCUGGGUUCUGAGCU | GTGCAGGGTCCGAGGTCAGAGCCACCTGGGCAATTTTTTTTTTTAGCTCA |
| 42 | >hsa-miR-4289 MIMAT0016920 | GTCGGGCATTGTGCAGGG | GCAUUGUGCAGGGCUAUCA | GTGCAGGGTCCGAGGTCAGAGCCACCTGGGCAATTTTTTTTTTTGATAGC |
| 42 | >hsa-miR-1537-3p MIMAT0007399 | TTCGGAAAACCGTCTAGTTAC | AAAACCGUCUAGUUACAGUUGU | GTGCAGGGTCCGAGGTCAGAGCCACCTGGGCAATTTTTTTTTTTACAACT |
| 42 | >hsa-miR-448 MIMAT0001532 | TTCGGTTGCATATGTAGGATG | UUGCAUAUGUAGGAUGUCCCAU | GTGCAGGGTCCGAGGTCAGAGCCACCTGGGCAATTTTTTTTTTTATGGGA |
| 43 | >hsa-let-7c-5p MIMAT0000064 | TCGGTGAGGTAGTAGGTTGT | UGAGGUAGUAGGUUGUAUGGUU | GTGCAGGGTCCGAGGTCAGAGCCACCTGGGCAATTTTTTTTTTTAACCAT |
| 43 | >hsa-miR-152-3p MIMAT0000438 | TTCGGTCAGTGCATGACAGA | UCAGUGCAUGACAGAACUUGG | GTGCAGGGTCCGAGGTCAGAGCCACCTGGGCAATTTTTTTTTTTCCAAGT |
| 43 | >hsa-miR-299-3p MIMAT0000687 | TTCGGTATGTGGGATGGTAAA | UAUGUGGGAUGGUAAACCGCUU | GTGCAGGGTCCGAGGTCAGAGCCACCTGGGCAATTTTTTTTTTTAAGCGG |
| 43 | >hsa-miR-214-3p MIMAT0000271 | TGGACAGCAGGCACAGACA | ACAGCAGGCACAGACAGGCAGU | GTGCAGGGTCCGAGGTCAGAGCCACCTGGGCAATTTTTTTTTTTACTGCC |
| 43 | >hsa-miR-4291 MIMAT0016922 | TTCGGTCGGTTCAGCAGGA | UUCAGCAGGAACAGCU | GTGCAGGGTCCGAGGTCAGAGCCACCTGGGCAATTTTTTTTTTTAGCTGT |
| 43 | >hsa-miR-2392 MIMAT0019043 | TTCGGTAGGATGGGGGTGA | UAGGAUGGGGGUGAGAGGUG | GTGCAGGGTCCGAGGTCAGAGCCACCTGGGCAATTTTTTTTTTTCACCTC |
| 43 | >hsa-miR-543 MIMAT0004954 | CGGAAACATTCGCGGTGCA | AAACAUUCGCGGUGCACUUCUU | GTGCAGGGTCCGAGGTCAGAGCCACCTGGGCAATTTTTTTTTTTAAGAAG |
| 44 | >hsa-miR-148a-5p MIMAT0004549 | TTCGGAAAGTTCTGAGACACT | AAAGUUCUGAGACACUCCGACU | GTGCAGGGTCCGAGGTCAGAGCCACCTGGGCAATTTTTTTTTTTAGTCGG |
| 44 | >hsa-let-7e-5p MIMAT0000066 | CGGTGAGGTAGGAGGTTGT | UGAGGUAGGAGGUUGUAUAGUU | GTGCAGGGTCCGAGGTCAGAGCCACCTGGGCAATTTTTTTTTTTAACTAT |
| 44 | >hsa-miR-3115 MIMAT0014977 | TGGTCGGATATGGGTTTACTA | AUAUGGGUUUACUAGUUGGU | GTGCAGGGTCCGAGGTCAGAGCCACCTGGGCAATTTTTTTTTTTACCAAC |
| 44 | >hsa-miR-3972 MIMAT0019357 | GCTGCCAGCCCCGTTCC | CUGCCAGCCCCGUUCCAGGGCA | GTGCAGGGTCCGAGGTCAGAGCCACCTGGGCAATTTTTTTTTTTGCCCTG |
| 44 | >hsa-miR-129-1-3p MIMAT0004548 | CGGAAGCCCTTACCCCAAA | AAGCCCUUACCCCAAAAAGUAU | GTGCAGGGTCCGAGGTCAGAGCCACCTGGGCAATTTTTTTTTTTATACTT |
| 44 | >hsa-miR-590-3p MIMAT0004801 | TCGGTCGGTAATTTTATGTATAA | UAAUUUUAUGUAUAAGCUAGU | GTGCAGGGTCCGAGGTCAGAGCCACCTGGGCAATTTTTTTTTTTACTAGC |
| 44 | >hsa-miR-454-5p MIMAT0003884 | GTCGGACCCTATCAATATTGT | ACCCUAUCAAUAUUGUCUCUGC | GTGCAGGGTCCGAGGTCAGAGCCACCTGGGCAATTTTTTTTTTTGCAGAG |
| 45 | >hsa-miR-196a-5p MIMAT0000226 | GTCGGTAGGTAGTTTCATGTT | UAGGUAGUUUCAUGUUGUUGGG | GTGCAGGGTCCGAGGTCAGAGCCACCTGGGCAATTTTTTTTTTTCCCAAC |
| 45 | >hsa-miR-4692 MIMAT0019783 | TGGTCAGGCAGTGTGGGTA | UCAGGCAGUGUGGGUAUCAGAU | GTGCAGGGTCCGAGGTCAGAGCCACCTGGGCAATTTTTTTTTTTATCTGA |
| 45 | >hsa-miR-3152-3p MIMAT0015025 | TCGGTGTGTTAGAATAGGGG | UGUGUUAGAAUAGGGGCAAUAA | GTGCAGGGTCCGAGGTCAGAGCCACCTGGGCAATTTTTTTTTTTATTGCC |
| 45 | >hsa-miR-150-5p MIMAT0000451 | CGGTCTCCCAACCCTTGTA | UCUCCCAACCCUUGUACCAGUG | GTGCAGGGTCCGAGGTCAGAGCCACCTGGGCAATTTTTTTTTTTCACTGG |
| 45 | >hsa-miR-622 MIMAT0003291 | TCGGACAGTCTGCTGAGGT | ACAGUCUGCUGAGGUUGGAGC | GTGCAGGGTCCGAGGTCAGAGCCACCTGGGCAATTTTTTTTTTTGCTCCA |
| 45 | >hsa-miR-3607-5p MIMAT0017984 | TCGGGCATGTGATGAAGCAA | GCAUGUGAUGAAGCAAAUCAGU | GTGCAGGGTCCGAGGTCAGAGCCACCTGGGCAATTTTTTTTTTTACTGAT |
| 45 | >hsa-miR-520a-5p MIMAT0002833 | TCGGCTCCAGAGGGAAGTA | CUCCAGAGGGAAGUACUUUCU | GTGCAGGGTCCGAGGTCAGAGCCACCTGGGCAATTTTTTTTTTTAGAAAG |
| 46 | >hsa-miR-361-5p MIMAT0000703 | TTCGGTTATCAGAATCTCCAG | UUAUCAGAAUCUCCAGGGGUAC | GTGCAGGGTCCGAGGTCAGAGCCACCTGGGCAATTTTTTTTTTTGTACCC |
| 46 | >hsa-miR-2116-3p MIMAT0011161 | CGGCCTCCCATGCCAAGA | CCUCCCAUGCCAAGAACUCCC | GTGCAGGGTCCGAGGTCAGAGCCACCTGGGCAATTTTTTTTTTTGGGAGT |
| 46 | >hsa-miR-551a MIMAT0003214 | TGGGCGACCCACTCTTGG | GCGACCCACUCUUGGUUUCCA | GTGCAGGGTCCGAGGTCAGAGCCACCTGGGCAATTTTTTTTTTTGGAAAC |
| 46 | >hsa-miR-454-3p MIMAT0003885 | TTCGGTAGTGCAATATTGCTTA | UAGUGCAAUAUUGCUUAUAGGGU | GTGCAGGGTCCGAGGTCAGAGCCACCTGGGCAATTTTTTTTTTTACCCTA |
| 46 | >hsa-miR-518a-3p MIMAT0002863 | CGGGAAAGCGCTTCCCTTT | GAAAGCGCUUCCCUUUGCUGGA | GTGCAGGGTCCGAGGTCAGAGCCACCTGGGCAATTTTTTTTTTTCCAGCA |
| 46 | >hsa-miR-3177-3p MIMAT0015054 | GGTGCACGGCACTGGGG | UGCACGGCACUGGGGACACGU | GTGCAGGGTCCGAGGTCAGAGCCACCTGGGCAATTTTTTTTTTTACGTGT |
| 46 | >hsa-miR-629-5p MIMAT0004810 | TCGGTGGGTTTACGTTGGG | UGGGUUUACGUUGGGAGAACU | GTGCAGGGTCCGAGGTCAGAGCCACCTGGGCAATTTTTTTTTTTAGTTCT |
| 47 | >hsa-miR-136-5p MIMAT0000448 | TTCGGACTCCATTTGTTTTGAT | ACUCCAUUUGUUUUGAUGAUGGA | GTGCAGGGTCCGAGGTCAGAGCCACCTGGGCAATTTTTTTTTTTCCATCA |
| 47 | >hsa-miR-1288 MIMAT0005942 | CGGTGGACTGCCCTGATC | UGGACUGCCCUGAUCUGGAGA | GTGCAGGGTCCGAGGTCAGAGCCACCTGGGCAATTTTTTTTTTTCTCCAG |
| 47 | >hsa-miR-576-3p MIMAT0004796 | TGTCGGAAGATGTGGAAAAATT | AAGAUGUGGAAAAAUUGGAAUC | GTGCAGGGTCCGAGGTCAGAGCCACCTGGGCAATTTTTTTTTTTGATTCC |
| 47 | >hsa-miR-4422 MIMAT0018935 | GTCGGAAAAGCATCAGGAAG | AAAAGCAUCAGGAAGUACCCA | GTGCAGGGTCCGAGGTCAGAGCCACCTGGGCAATTTTTTTTTTTGGGTAC |
| 47 | >hsa-miR-483-5p MIMAT0004761 | CGGAAGACGGGAGGAAAGA | AAGACGGGAGGAAAGAAGGGAG | GTGCAGGGTCCGAGGTCAGAGCCACCTGGGCAATTTTTTTTTTTCTCCCT |
| 47 | >hsa-miR-766-3p MIMAT0003888 | TGACTCCAGCCCCACAGC | ACUCCAGCCCCACAGCCUCAGC | GTGCAGGGTCCGAGGTCAGAGCCACCTGGGCAATTTTTTTTTTTGCTGAG |
| 47 | >hsa-miR-30c-2-3p MIMAT0004550 | TGGCTGGGAGAAGGCTGTT | CUGGGAGAAGGCUGUUUACUCU | GTGCAGGGTCCGAGGTCAGAGCCACCTGGGCAATTTTTTTTTTTAGAGTA |
| 48 | >hsa-miR-200b-3p MIMAT0000318 | TTCGGTAATACTGCCTGGTAA | UAAUACUGCCUGGUAAUGAUGA | GTGCAGGGTCCGAGGTCAGAGCCACCTGGGCAATTTTTTTTTTTCATCAT |
| 48 | >hsa-miR-183-3p MIMAT0004560 | CGGGTGAATTACCGAAGGG | GUGAAUUACCGAAGGGCCAUAA | GTGCAGGGTCCGAGGTCAGAGCCACCTGGGCAATTTTTTTTTTTATGGCC |
| 48 | >hsa-miR-532-3p MIMAT0004780 | TGCCTCCCACACCCAAGG | CCUCCCACACCCAAGGCUUGCA | GTGCAGGGTCCGAGGTCAGAGCCACCTGGGCAATTTTTTTTTTTGCAAGC |
| 48 | >hsa-miR-1265 MIMAT0005918 | CGGCAGGATGTGGTCAAGT | CAGGAUGUGGUCAAGUGUUGUU | GTGCAGGGTCCGAGGTCAGAGCCACCTGGGCAATTTTTTTTTTTAACAAC |
| 48 | >hsa-miR-204-5p MIMAT0000265 | TCGGTTCCCTTTGTCATCCT | UUCCCUUUGUCAUCCUAUGCCU | GTGCAGGGTCCGAGGTCAGAGCCACCTGGGCAATTTTTTTTTTTAGGCAT |
| 48 | >hsa-miR-367-3p MIMAT0000719 | GTCGGAATTGCACTTTAGCAA | AAUUGCACUUUAGCAAUGGUGA | GTGCAGGGTCCGAGGTCAGAGCCACCTGGGCAATTTTTTTTTTTCACCAT |
| 48 | >hsa-miR-101-5p MIMAT0004513 | TCGGCAGTTATCACAGTGCT | CAGUUAUCACAGUGCUGAUGCU | GTGCAGGGTCCGAGGTCAGAGCCACCTGGGCAATTTTTTTTTTTAGCATC |
| 49 | >hsa-miR-196b-5p MIMAT0001080 | TTCGGTAGGTAGTTTCCTGTT | UAGGUAGUUUCCUGUUGUUGGG | GTGCAGGGTCCGAGGTCAGAGCCACCTGGGCAATTTTTTTTTTTCCCAAC |
| 49 | >hsa-miR-550a-3p MIMAT0003257 | CGGTGTCTTACTCCCTCAG | UGUCUUACUCCCUCAGGCACAU | GTGCAGGGTCCGAGGTCAGAGCCACCTGGGCAATTTTTTTTTTTATGTGC |
| 49 | >hsa-miR-186-5p MIMAT0000456 | GTCGGCAAAGAATTCTCCTTT | CAAAGAAUUCUCCUUUUGGGCU | GTGCAGGGTCCGAGGTCAGAGCCACCTGGGCAATTTTTTTTTTTAGCCCA |
| 49 | >hsa-miR-425-5p MIMAT0003393 | TGGAATGACACGATCACTCC | AAUGACACGAUCACUCCCGUUGA | GTGCAGGGTCCGAGGTCAGAGCCACCTGGGCAATTTTTTTTTTTCAACGG |
| 49 | >hsa-miR-218-5p MIMAT0000275 | TGTCGGTTGTGCTTGATCTAA | UUGUGCUUGAUCUAACCAUGU | GTGCAGGGTCCGAGGTCAGAGCCACCTGGGCAATTTTTTTTTTTACATGG |
| 49 | >hsa-miR-379-5p MIMAT0000733 | GTCGGTGGTAGACTATGGAA | UGGUAGACUAUGGAACGUAGG | GTGCAGGGTCCGAGGTCAGAGCCACCTGGGCAATTTTTTTTTTTCCTACG |
| 49 | >hsa-miR-449c-5p MIMAT0010251 | GTAGGCAGTGTATTGCTAGC | UAGGCAGUGUAUUGCUAGCGGCUGU | GTGCAGGGTCCGAGGTCAGAGCCACCTGGGCAATTTTTTTTTTTACAGCC |
| 50 | >hsa-miR-3074-3p MIMAT0015027 | TCGGGATATCAGCTCAGTAG | GAUAUCAGCUCAGUAGGCACCG | GTGCAGGGTCCGAGGTCAGAGCCACCTGGGCAATTTTTTTTTTTCGGTGC |
| 50 | >hsa-miR-133a-3p MIMAT0000427 | CGGTTTGGTCCCCTTCAAC | UUUGGUCCCCUUCAACCAGCUG | GTGCAGGGTCCGAGGTCAGAGCCACCTGGGCAATTTTTTTTTTTCAGCTG |
| 50 | >hsa-miR-1277-5p MIMAT0022724 | GGTCGGAAATATATATATATATGT | AAAUAUAUAUAUAUAUGUACGUAU | GTGCAGGGTCCGAGGTCAGAGCCACCTGGGCAATTTTTTTTTTTATACGT |
| 50 | >hsa-miR-106b-3p MIMAT0004672 | GGCCGCACTGTGGGTACT | CCGCACUGUGGGUACUUGCUGC | GTGCAGGGTCCGAGGTCAGAGCCACCTGGGCAATTTTTTTTTTTGCAGCA |
| 50 | >hsa-miR-145-3p MIMAT0004601 | TCGGGGATTCCTGGAAATAC | GGAUUCCUGGAAAUACUGUUCU | GTGCAGGGTCCGAGGTCAGAGCCACCTGGGCAATTTTTTTTTTTAGAACA |
| 50 | >hsa-miR-487b-3p MIMAT0003180 | TCGGAATCGTACAGGGTCAT | AAUCGUACAGGGUCAUCCACUU | GTGCAGGGTCCGAGGTCAGAGCCACCTGGGCAATTTTTTTTTTTAAGTGG |
| 50 | >hsa-miR-10b-3p MIMAT0004556 | TTCGGACAGATTCGATTCTAG | ACAGAUUCGAUUCUAGGGGAAU | GTGCAGGGTCCGAGGTCAGAGCCACCTGGGCAATTTTTTTTTTTATTCCC |
| 51 | >hsa-miR-3935 MIMAT0018350 | CGGTGTAGATACGAGCACC | UGUAGAUACGAGCACCAGCCAC | GTGCAGGGTCCGAGGTCAGAGCCACCTGGGCAATTTTTTTTTTTGTGGCT |
| 51 | >hsa-let-7g-5p MIMAT0000414 | TTCGGTGAGGTAGTAGTTTGT | UGAGGUAGUAGUUUGUACAGUU | GTGCAGGGTCCGAGGTCAGAGCCACCTGGGCAATTTTTTTTTTTAACTGT |
| 51 | >hsa-miR-1255b-2-3p MIMAT0022725 | TTCGGAACCACTTTCTTTGCT | AACCACUUUCUUUGCUCAUCCA | GTGCAGGGTCCGAGGTCAGAGCCACCTGGGCAATTTTTTTTTTTGGATGA |
| 51 | >hsa-miR-527 MIMAT0002862 | TTCGGCTGCAAAGGGAAGC | CUGCAAAGGGAAGCCCUUUC | GTGCAGGGTCCGAGGTCAGAGCCACCTGGGCAATTTTTTTTTTTGAAAGG |
| 51 | >hsa-miR-450a-3p MIMAT0022700 | TCGGATTGGGGACATTTTGC | AUUGGGGACAUUUUGCAUUCAU | GTGCAGGGTCCGAGGTCAGAGCCACCTGGGCAATTTTTTTTTTTATGAAT |
| 51 | >hsa-miR-499a-5p MIMAT0002870 | GTCGGTTAAGACTTGCAGTG | UUAAGACUUGCAGUGAUGUUU | GTGCAGGGTCCGAGGTCAGAGCCACCTGGGCAATTTTTTTTTTTAAACAT |
| 51 | >hsa-miR-17-5p MIMAT0000070 | TGGCAAAGTGCTTACAGTGC | CAAAGUGCUUACAGUGCAGGUAG | GTGCAGGGTCCGAGGTCAGAGCCACCTGGGCAATTTTTTTTTTTCTACCT |
| 52 | >hsa-miR-1913 MIMAT0007888 | GTCTGCCCCCTCCGCTG | UCUGCCCCCUCCGCUGCUGCCA | GTGCAGGGTCCGAGGTCAGAGCCACCTGGGCAATTTTTTTTTTTGGCAGC |
| 52 | >hsa-miR-4732-5p MIMAT0019855 | TGTGTAGAGCAGGGAGCAG | UGUAGAGCAGGGAGCAGGAAGCU | GTGCAGGGTCCGAGGTCAGAGCCACCTGGGCAATTTTTTTTTTTAGCTTC |
| 52 | >hsa-miR-660-5p MIMAT0003338 | TCGGTACCCATTGCATATCG | UACCCAUUGCAUAUCGGAGUUG | GTGCAGGGTCCGAGGTCAGAGCCACCTGGGCAATTTTTTTTTTTCAACTC |
| 52 | >hsa-miR-26a-1-3p MIMAT0004499 | TTCGGCCTATTCTTGGTTACT | CCUAUUCUUGGUUACUUGCACG | GTGCAGGGTCCGAGGTCAGAGCCACCTGGGCAATTTTTTTTTTTCGTGCA |
| 52 | >hsa-miR-3941 MIMAT0018357 | TCGGTTACACACAACTGAGG | UUACACACAACUGAGGAUCAUA | GTGCAGGGTCCGAGGTCAGAGCCACCTGGGCAATTTTTTTTTTTATGATC |
| 52 | >hsa-miR-624-5p MIMAT0003293 | TCGGTAGTACCAGTACCTTG | UAGUACCAGUACCUUGUGUUCA | GTGCAGGGTCCGAGGTCAGAGCCACCTGGGCAATTTTTTTTTTTGAACAC |
| 52 | >hsa-miR-28-3p MIMAT0004502 | TCGGCACTAGATTGTGAGCT | CACUAGAUUGUGAGCUCCUGGA | GTGCAGGGTCCGAGGTCAGAGCCACCTGGGCAATTTTTTTTTTTCCAGGA |
| 53 | >hsa-miR-1290 MIMAT0005880 | CGGTCGGTGGATTTTTGGAT | UGGAUUUUUGGAUCAGGGA | GTGCAGGGTCCGAGGTCAGAGCCACCTGGGCAATTTTTTTTTTTCCCTGA |
| 53 | >hsa-miR-93-5p MIMAT0000093 | GGCAAAGTGCTGTTCGTGC | CAAAGUGCUGUUCGUGCAGGUAG | GTGCAGGGTCCGAGGTCAGAGCCACCTGGGCAATTTTTTTTTTTCTACCT |
| 53 | >hsa-miR-455-3p MIMAT0004784 | CGGGCAGTCCATGGGCAT | GCAGUCCAUGGGCAUAUACAC | GTGCAGGGTCCGAGGTCAGAGCCACCTGGGCAATTTTTTTTTTTGTGTAT |
| 53 | >hsa-miR-98-5p MIMAT0000096 | TTCGGTGAGGTAGTAAGTTGT | UGAGGUAGUAAGUUGUAUUGUU | GTGCAGGGTCCGAGGTCAGAGCCACCTGGGCAATTTTTTTTTTTAACAAT |
| 53 | >hsa-miR-518e-5p MIMAT0005450 | GGCTCTAGAGGGAAGCGC | CUCUAGAGGGAAGCGCUUUCUG | GTGCAGGGTCCGAGGTCAGAGCCACCTGGGCAATTTTTTTTTTTCAGAAA |
| 53 | >hsa-miR-539-5p MIMAT0003163 | TTCGGGGAGAAATTATCCTTG | GGAGAAAUUAUCCUUGGUGUGU | GTGCAGGGTCCGAGGTCAGAGCCACCTGGGCAATTTTTTTTTTTACACAC |
| 53 | >hsa-miR-148a-3p MIMAT0000243 | TCGGTCAGTGCACTACAGAA | UCAGUGCACUACAGAACUUUGU | GTGCAGGGTCCGAGGTCAGAGCCACCTGGGCAATTTTTTTTTTTACAAAG |
| 54 | >hsa-miR-26b-5p MIMAT0000083 | TGTCGGTTCAAGTAATTCAGG | UUCAAGUAAUUCAGGAUAGGU | GTGCAGGGTCCGAGGTCAGAGCCACCTGGGCAATTTTTTTTTTTACCTAT |
| 54 | >hsa-miR-2276-3p MIMAT0011775 | CGGTCTGCAAGTGTCAGAG | UCUGCAAGUGUCAGAGGCGAGG | GTGCAGGGTCCGAGGTCAGAGCCACCTGGGCAATTTTTTTTTTTCCTCGC |
| 54 | >hsa-miR-28-5p MIMAT0000085 | CGGAAGGAGCTCACAGTCT | AAGGAGCUCACAGUCUAUUGAG | GTGCAGGGTCCGAGGTCAGAGCCACCTGGGCAATTTTTTTTTTTCTCAAT |
| 54 | >hsa-miR-516a-3p MIMAT0006778 | CGGTCGGTGCTTCCTTTCA | UGCUUCCUUUCAGAGGGU | GTGCAGGGTCCGAGGTCAGAGCCACCTGGGCAATTTTTTTTTTTACCCTC |
| 54 | >hsa-miR-7-5p MIMAT0000252 | TCGGTGGAAGACTAGTGATTT | UGGAAGACUAGUGAUUUUGUUGU | GTGCAGGGTCCGAGGTCAGAGCCACCTGGGCAATTTTTTTTTTTACAACA |
| 54 | >hsa-miR-29a-3p MIMAT0000086 | TTCGGTAGCACCATCTGAAAT | UAGCACCAUCUGAAAUCGGUUA | GTGCAGGGTCCGAGGTCAGAGCCACCTGGGCAATTTTTTTTTTTAACCGA |
| 54 | >hsa-miR-129-2-3p MIMAT0004605 | CGGAAGCCCTTACCCCAAA | AAGCCCUUACCCCAAAAAGCAU | GTGCAGGGTCCGAGGTCAGAGCCACCTGGGCAATTTTTTTTTTTATGCTT |
| 55 | >hsa-miR-3128 MIMAT0014991 | TCGGTCTGGCAAGTAAAAAAC | UCUGGCAAGUAAAAAACUCUCAU | GTGCAGGGTCCGAGGTCAGAGCCACCTGGGCAATTTTTTTTTTTATGAGA |
| 55 | >hsa-miR-101-3p MIMAT0000099 | TGTCGGTACAGTACTGTGATA | UACAGUACUGUGAUAACUGAA | GTGCAGGGTCCGAGGTCAGAGCCACCTGGGCAATTTTTTTTTTTCAGTTA |
| 55 | >hsa-miR-32-5p MIMAT0000090 | TGTCGGTATTGCACATTACTAA | UAUUGCACAUUACUAAGUUGCA | GTGCAGGGTCCGAGGTCAGAGCCACCTGGGCAATTTTTTTTTTTGCAACT |
| 55 | >hsa-miR-143-3p MIMAT0000435 | TTCGGTGAGATGAAGCACTG | UGAGAUGAAGCACUGUAGCUC | GTGCAGGGTCCGAGGTCAGAGCCACCTGGGCAATTTTTTTTTTTGAGCTA |
| 55 | >hsa-miR-187-3p MIMAT0000262 | CGGTCGTGTCTTGTGTTGC | UCGUGUCUUGUGUUGCAGCCGG | GTGCAGGGTCCGAGGTCAGAGCCACCTGGGCAATTTTTTTTTTTCCGGCT |
| 55 | >hsa-miR-3180-5p MIMAT0015057 | CTTCCAGACGCTCCGCCCC | CUUCCAGACGCUCCGCCCCACGUCG | GTGCAGGGTCCGAGGTCAGAGCCACCTGGGCAATTTTTTTTTTTCGACGT |
| 55 | >hsa-miR-517-5p MIMAT0002851 | CGGCCTCTAGATGGAAGCA | CCUCUAGAUGGAAGCACUGUCU | GTGCAGGGTCCGAGGTCAGAGCCACCTGGGCAATTTTTTTTTTTAGACAG |
| 56 | >hsa-miR-141-3p MIMAT0000432 | TTCGGTAACACTGTCTGGTAA | UAACACUGUCUGGUAAAGAUGG | GTGCAGGGTCCGAGGTCAGAGCCACCTGGGCAATTTTTTTTTTTCCATCT |
| 56 | >hsa-miR-4423-5p MIMAT0019232 | TTCGGAGTTGCCTTTTTGTTC | AGUUGCCUUUUUGUUCCCAUGC | GTGCAGGGTCCGAGGTCAGAGCCACCTGGGCAATTTTTTTTTTTGCATGG |
| 56 | >hsa-miR-181a-3p MIMAT0000270 | CGGACCATCGACCGTTGAT | ACCAUCGACCGUUGAUUGUACC | GTGCAGGGTCCGAGGTCAGAGCCACCTGGGCAATTTTTTTTTTTGGTACA |
| 56 | >hsa-let-7i-5p MIMAT0000415 | TTCGGTGAGGTAGTAGTTTGT | UGAGGUAGUAGUUUGUGCUGUU | GTGCAGGGTCCGAGGTCAGAGCCACCTGGGCAATTTTTTTTTTTAACAGC |
| 56 | >hsa-miR-3130-5p MIMAT0014995 | CGGTACCCAGTCTCCGGT | UACCCAGUCUCCGGUGCAGCC | GTGCAGGGTCCGAGGTCAGAGCCACCTGGGCAATTTTTTTTTTTGGCTGC |
| 56 | >hsa-miR-106a-5p MIMAT0000103 | CGGAAAAGTGCTTACAGTGC | AAAAGUGCUUACAGUGCAGGUAG | GTGCAGGGTCCGAGGTCAGAGCCACCTGGGCAATTTTTTTTTTTCTACCT |
| 56 | >hsa-miR-296-5p MIMAT0000690 | GGAGGGCCCCCCCTCAA | AGGGCCCCCCCUCAAUCCUGU | GTGCAGGGTCCGAGGTCAGAGCCACCTGGGCAATTTTTTTTTTTACAGGA |
| 57 | >hsa-miR-374a-3p MIMAT0004688 | TGTCGGCTTATCAGATTGTATT | CUUAUCAGAUUGUAUUGUAAUU | GTGCAGGGTCCGAGGTCAGAGCCACCTGGGCAATTTTTTTTTTTAATTAC |
| 57 | >hsa-miR-216a-5p MIMAT0000273 | TCGGTAATCTCAGCTGGCAA | UAAUCUCAGCUGGCAACUGUGA | GTGCAGGGTCCGAGGTCAGAGCCACCTGGGCAATTTTTTTTTTTCACAGT |
| 57 | >hsa-miR-518f-3p MIMAT0002842 | TTCGGGAAAGCGCTTCTCTT | GAAAGCGCUUCUCUUUAGAGG | GTGCAGGGTCCGAGGTCAGAGCCACCTGGGCAATTTTTTTTTTTCCTCTA |
| 57 | >hsa-miR-106b-5p MIMAT0000680 | GTCGGTAAAGTGCTGACAGT | UAAAGUGCUGACAGUGCAGAU | GTGCAGGGTCCGAGGTCAGAGCCACCTGGGCAATTTTTTTTTTTATCTGC |
| 57 | >hsa-miR-744-5p MIMAT0004945 | TGTGCGGGGCTAGGGCTA | UGCGGGGCUAGGGCUAACAGCA | GTGCAGGGTCCGAGGTCAGAGCCACCTGGGCAATTTTTTTTTTTGCTGTT |
| 57 | >hsa-miR-144-3p MIMAT0000436 | TGGTCGGTACAGTATAGATGA | UACAGUAUAGAUGAUGUACU | GTGCAGGGTCCGAGGTCAGAGCCACCTGGGCAATTTTTTTTTTTAGTACA |
| 57 | >hsa-miR-509-3-5p MIMAT0004975 | TGGTACTGCAGACGTGGCA | UACUGCAGACGUGGCAAUCAUG | GTGCAGGGTCCGAGGTCAGAGCCACCTGGGCAATTTTTTTTTTTCATGAT |
| 58 | >hsa-miR-3065-5p MIMAT0015066 | TTCGGTCAACAAAATCACTGAT | UCAACAAAAUCACUGAUGCUGGA | GTGCAGGGTCCGAGGTCAGAGCCACCTGGGCAATTTTTTTTTTTCCAGCA |
| 58 | >hsa-miR-192-5p MIMAT0000222 | GTCGGCTGACCTATGAATTG | CUGACCUAUGAAUUGACAGCC | GTGCAGGGTCCGAGGTCAGAGCCACCTGGGCAATTTTTTTTTTTGGCTGT |
| 58 | >hsa-miR-29a-5p MIMAT0004503 | GTCGGACTGATTTCTTTTGGT | ACUGAUUUCUUUUGGUGUUCAG | GTGCAGGGTCCGAGGTCAGAGCCACCTGGGCAATTTTTTTTTTTCTGAAC |
| 58 | >hsa-miR-345-5p MIMAT0000772 | TGGGCTGACTCCTAGTCCA | GCUGACUCCUAGUCCAGGGCUC | GTGCAGGGTCCGAGGTCAGAGCCACCTGGGCAATTTTTTTTTTTGAGCCC |
| 58 | >hsa-miR-411-5p MIMAT0003329 | GTCGGTAGTAGACCGTATAG | UAGUAGACCGUAUAGCGUACG | GTGCAGGGTCCGAGGTCAGAGCCACCTGGGCAATTTTTTTTTTTCGTACG |
| 58 | >hsa-let-7i-3p MIMAT0004585 | GGCTGCGCAAGCTACTGC | CUGCGCAAGCUACUGCCUUGCU | GTGCAGGGTCCGAGGTCAGAGCCACCTGGGCAATTTTTTTTTTTAGCAAG |
| 58 | >hsa-miR-146b-5p MIMAT0002809 | TTCGGTGAGAACTGAATTCCA | UGAGAACUGAAUUCCAUAGGCU | GTGCAGGGTCCGAGGTCAGAGCCACCTGGGCAATTTTTTTTTTTAGCCTA |
| 59 | >hsa-miR-19a-3p MIMAT0000073 | TCGGTGTGCAAATCTATGCAA | UGUGCAAAUCUAUGCAAAACUGA | GTGCAGGGTCCGAGGTCAGAGCCACCTGGGCAATTTTTTTTTTTCAGTTT |
| 59 | >hsa-miR-99a-5p MIMAT0000097 | CGGAACCCGTAGATCCGAT | AACCCGUAGAUCCGAUCUUGUG | GTGCAGGGTCCGAGGTCAGAGCCACCTGGGCAATTTTTTTTTTTCACAAG |
| 59 | >hsa-miR-503-5p MIMAT0002874 | GGTAGCAGCGGGAACAGTT | UAGCAGCGGGAACAGUUCUGCAG | GTGCAGGGTCCGAGGTCAGAGCCACCTGGGCAATTTTTTTTTTTCTGCAG |
| 59 | >hsa-miR-23a-3p MIMAT0000078 | TCGGATCACATTGCCAGGG | AUCACAUUGCCAGGGAUUUCC | GTGCAGGGTCCGAGGTCAGAGCCACCTGGGCAATTTTTTTTTTTGGAAAT |
| 59 | >hsa-miR-20b-5p MIMAT0001413 | TGGCAAAGTGCTCATAGTGC | CAAAGUGCUCAUAGUGCAGGUAG | GTGCAGGGTCCGAGGTCAGAGCCACCTGGGCAATTTTTTTTTTTCTACCT |
| 59 | >hsa-miR-378a-3p MIMAT0000732 | CGGACTGGACTTGGAGTCA | ACUGGACUUGGAGUCAGAAGGC | GTGCAGGGTCCGAGGTCAGAGCCACCTGGGCAATTTTTTTTTTTGCCTTC |
| 59 | >hsa-miR-151b MIMAT0010214 | GGTCGGTCGAGGAGCTCA | UCGAGGAGCUCACAGUCU | GTGCAGGGTCCGAGGTCAGAGCCACCTGGGCAATTTTTTTTTTTAGACTG |
| 60 | >hsa-miR-1255b-5p MIMAT0005945 | TCGGCGGATGAGCAAAGAAA | CGGAUGAGCAAAGAAAGUGGUU | GTGCAGGGTCCGAGGTCAGAGCCACCTGGGCAATTTTTTTTTTTAACCAC |
| 60 | >hsa-miR-519c-3p MIMAT0002832 | TGTCGGAAAGTGCATCTTTTTA | AAAGUGCAUCUUUUUAGAGGAU | GTGCAGGGTCCGAGGTCAGAGCCACCTGGGCAATTTTTTTTTTTATCCTC |
| 60 | >hsa-miR-16-1-3p MIMAT0004489 | TCGGCCAGTATTAACTGTGC | CCAGUAUUAACUGUGCUGCUGA | GTGCAGGGTCCGAGGTCAGAGCCACCTGGGCAATTTTTTTTTTTCAGCAG |
| 60 | >hsa-miR-320d MIMAT0006764 | GGTCGGAAAAGCTGGGTTG | AAAAGCUGGGUUGAGAGGA | GTGCAGGGTCCGAGGTCAGAGCCACCTGGGCAATTTTTTTTTTTCCTCTC |
| 60 | >hsa-miR-2355-5p MIMAT0016895 | GTCGGATCCCCAGATACAAT | AUCCCCAGAUACAAUGGACAA | GTGCAGGGTCCGAGGTCAGAGCCACCTGGGCAATTTTTTTTTTTGTCCAT |
| 60 | >hsa-miR-345-3p MIMAT0022698 | TGGCCCTGAACGAGGGGT | GCCCUGAACGAGGGGUCUGGAG | GTGCAGGGTCCGAGGTCAGAGCCACCTGGGCAATTTTTTTTTTTCTCCAG |
| 60 | >hsa-miR-30e-5p MIMAT0000692 | TTCGGTGTAAACATCCTTGAC | UGUAAACAUCCUUGACUGGAAG | GTGCAGGGTCCGAGGTCAGAGCCACCTGGGCAATTTTTTTTTTTCTTCCA |
| 61 | >hsa-miR-487a-3p MIMAT0002178 | TTCGGAATCATACAGGGACAT | AAUCAUACAGGGACAUCCAGUU | GTGCAGGGTCCGAGGTCAGAGCCACCTGGGCAATTTTTTTTTTTAACTGG |
| 61 | >hsa-miR-222-3p MIMAT0000279 | TTCGGAGCTACATCTGGCTA | AGCUACAUCUGGCUACUGGGU | GTGCAGGGTCCGAGGTCAGAGCCACCTGGGCAATTTTTTTTTTTACCCAG |
| 61 | >hsa-miR-548aw MIMAT0022471 | TGTCGGGTGCAAAAGTCATC | GUGCAAAAGUCAUCACGGUU | GTGCAGGGTCCGAGGTCAGAGCCACCTGGGCAATTTTTTTTTTTAACCGT |
| 61 | >hsa-miR-34c-5p MIMAT0000686 | TGGAGGCAGTGTAGTTAGCT | AGGCAGUGUAGUUAGCUGAUUGC | GTGCAGGGTCCGAGGTCAGAGCCACCTGGGCAATTTTTTTTTTTGCAATC |
| 61 | >hsa-miR-378g MIMAT0018937 | TTCGGACTGGGCTTGGAGT | ACUGGGCUUGGAGUCAGAAG | GTGCAGGGTCCGAGGTCAGAGCCACCTGGGCAATTTTTTTTTTTCTTCTG |
| 61 | >hsa-miR-1301-3p MIMAT0005797 | TTTGCAGCTGCCTGGGAGT | UUGCAGCUGCCUGGGAGUGACUUC | GTGCAGGGTCCGAGGTCAGAGCCACCTGGGCAATTTTTTTTTTTGAAGTC |
| 61 | >hsa-miR-2115-5p MIMAT0011158 | CGGAGCTTCCATGACTCCT | AGCUUCCAUGACUCCUGAUGGA | GTGCAGGGTCCGAGGTCAGAGCCACCTGGGCAATTTTTTTTTTTCCATCA |
| 62 | >hsa-miR-505-3p MIMAT0002876 | TGGCGTCAACACTTGCTGG | CGUCAACACUUGCUGGUUUCCU | GTGCAGGGTCCGAGGTCAGAGCCACCTGGGCAATTTTTTTTTTTAGGAAA |
| 62 | >hsa-miR-191-5p MIMAT0000440 | TGGCAACGGAATCCCAAAAG | CAACGGAAUCCCAAAAGCAGCUG | GTGCAGGGTCCGAGGTCAGAGCCACCTGGGCAATTTTTTTTTTTCAGCTG |
| 62 | >hsa-miR-218-2-3p MIMAT0004566 | CGGCATGGTTCTGTCAAGC | CAUGGUUCUGUCAAGCACCGCG | GTGCAGGGTCCGAGGTCAGAGCCACCTGGGCAATTTTTTTTTTTCGCGGT |
| 62 | >hsa-miR-3609 MIMAT0017986 | CGGCAAAGTGATGAGTAATAC | CAAAGUGAUGAGUAAUACUGGCUG | GTGCAGGGTCCGAGGTCAGAGCCACCTGGGCAATTTTTTTTTTTCAGCCA |
| 62 | >hsa-miR-618 MIMAT0003287 | TCGGAAACTCTACTTGTCCTT | AAACUCUACUUGUCCUUCUGAGU | GTGCAGGGTCCGAGGTCAGAGCCACCTGGGCAATTTTTTTTTTTACTCAG |
| 62 | >hsa-miR-190a-5p MIMAT0000458 | GGTCGGTGATATGTTTGATATA | UGAUAUGUUUGAUAUAUUAGGU | GTGCAGGGTCCGAGGTCAGAGCCACCTGGGCAATTTTTTTTTTTACCTAA |
| 62 | >hsa-miR-146a-3p MIMAT0004608 | TTCGGCCTCTGAAATTCAGTT | CCUCUGAAAUUCAGUUCUUCAG | GTGCAGGGTCCGAGGTCAGAGCCACCTGGGCAATTTTTTTTTTTCTGAAG |
| 63 | >hsa-miR-1469 MIMAT0007347 | CTCGGCGCGGGGCGCG | CUCGGCGCGGGGCGCGGGCUCC | GTGCAGGGTCCGAGGTCAGAGCCACCTGGGCAATTTTTTTTTTTGGAGCC |
| 63 | >hsa-miR-181d-5p MIMAT0002821 | TCGGAACATTCATTGTTGTCG | AACAUUCAUUGUUGUCGGUGGGU | GTGCAGGGTCCGAGGTCAGAGCCACCTGGGCAATTTTTTTTTTTACCCAC |
| 63 | >hsa-miR-491-5p MIMAT0002807 | GGAGTGGGGAACCCTTCC | AGUGGGGAACCCUUCCAUGAGG | GTGCAGGGTCCGAGGTCAGAGCCACCTGGGCAATTTTTTTTTTTCCTCAT |
| 63 | >hsa-miR-486-5p MIMAT0002177 | TGGTCCTGTACTGAGCTGC | UCCUGUACUGAGCUGCCCCGAG | GTGCAGGGTCCGAGGTCAGAGCCACCTGGGCAATTTTTTTTTTTCTCGGG |
| 63 | >hsa-miR-483-3p MIMAT0002173 | CGGTCACTCCTCTCCTCC | UCACUCCUCUCCUCCCGUCUU | GTGCAGGGTCCGAGGTCAGAGCCACCTGGGCAATTTTTTTTTTTAAGACG |
| 63 | >hsa-miR-26b-3p MIMAT0004500 | TCGGCCTGTTCTCCATTACT | CCUGUUCUCCAUUACUUGGCUC | GTGCAGGGTCCGAGGTCAGAGCCACCTGGGCAATTTTTTTTTTTGAGCCA |
| 63 | >hsa-miR-197-3p MIMAT0000227 | CGGTTCACCACCTTCTCCA | UUCACCACCUUCUCCACCCAGC | GTGCAGGGTCCGAGGTCAGAGCCACCTGGGCAATTTTTTTTTTTGCTGGG |
| 64 | >hsa-miR-571 MIMAT0003236 | TCGGTGAGTTGGCCATCTG | UGAGUUGGCCAUCUGAGUGAG | GTGCAGGGTCCGAGGTCAGAGCCACCTGGGCAATTTTTTTTTTTCTCACT |
| 64 | >hsa-miR-1197 MIMAT0005955 | TTCGGTAGGACACATGGTCT | UAGGACACAUGGUCUACUUCU | GTGCAGGGTCCGAGGTCAGAGCCACCTGGGCAATTTTTTTTTTTAGAAGT |
| 64 | >hsa-miR-126-5p MIMAT0000444 | GGTCGGCATTATTACTTTTGG | CAUUAUUACUUUUGGUACGCG | GTGCAGGGTCCGAGGTCAGAGCCACCTGGGCAATTTTTTTTTTTCGCGTA |
| 64 | >hsa-miR-320a MIMAT0000510 | TCGGAAAAGCTGGGTTGAGA | AAAAGCUGGGUUGAGAGGGCGA | GTGCAGGGTCCGAGGTCAGAGCCACCTGGGCAATTTTTTTTTTTCGCCCT |
| 64 | >hsa-miR-130b-3p MIMAT0000691 | TTCGGCAGTGCAATGATGAAA | CAGUGCAAUGAUGAAAGGGCAU | GTGCAGGGTCCGAGGTCAGAGCCACCTGGGCAATTTTTTTTTTTATGCCC |
| 64 | >hsa-miR-18a-5p MIMAT0000072 | TGGTAAGGTGCATCTAGTGC | UAAGGUGCAUCUAGUGCAGAUAG | GTGCAGGGTCCGAGGTCAGAGCCACCTGGGCAATTTTTTTTTTTCTATCT |
| 64 | >hsa-miR-654-5p MIMAT0003330 | TGTGGTGGGCCGCAGAAC | UGGUGGGCCGCAGAACAUGUGC | GTGCAGGGTCCGAGGTCAGAGCCACCTGGGCAATTTTTTTTTTTGCACAT |
| 65 | >hsa-miR-22-5p MIMAT0004495 | TCGGAGTTCTTCAGTGGCAA | AGUUCUUCAGUGGCAAGCUUUA | GTGCAGGGTCCGAGGTCAGAGCCACCTGGGCAATTTTTTTTTTTAAAGCT |
| 65 | >hsa-miR-548q MIMAT0011163 | TCGGGCTGGTGCAAAAGTAA | GCUGGUGCAAAAGUAAUGGCGG | GTGCAGGGTCCGAGGTCAGAGCCACCTGGGCAATTTTTTTTTTTCCGCCA |
| 65 | >hsa-miR-338-3p MIMAT0000763 | TCGGTCCAGCATCAGTGATT | UCCAGCAUCAGUGAUUUUGUUG | GTGCAGGGTCCGAGGTCAGAGCCACCTGGGCAATTTTTTTTTTTCAACAA |
| 65 | >hsa-miR-146a-5p MIMAT0000449 | TTCGGTGAGAACTGAATTCCA | UGAGAACUGAAUUCCAUGGGUU | GTGCAGGGTCCGAGGTCAGAGCCACCTGGGCAATTTTTTTTTTTAACCCA |
| 65 | >hsa-miR-363-3p MIMAT0000707 | TCGGAATTGCACGGTATCCA | AAUUGCACGGUAUCCAUCUGUA | GTGCAGGGTCCGAGGTCAGAGCCACCTGGGCAATTTTTTTTTTTACAGAT |
| 65 | >hsa-miR-1343 MIMAT0019776 | TCTCCTGGGGCCCGCAC | CUCCUGGGGCCCGCACUCUCGC | GTGCAGGGTCCGAGGTCAGAGCCACCTGGGCAATTTTTTTTTTTGCGAGA |
| 65 | >hsa-miR-342-3p MIMAT0000753 | TGGTCTCACACAGAAATCGC | UCUCACACAGAAAUCGCACCCGU | GTGCAGGGTCCGAGGTCAGAGCCACCTGGGCAATTTTTTTTTTTACGGGT |
| 66 | >hsa-miR-3129-5p MIMAT0014992 | TCGGGCAGTAGTGTAGAGAT | GCAGUAGUGUAGAGAUUGGUUU | GTGCAGGGTCCGAGGTCAGAGCCACCTGGGCAATTTTTTTTTTTAAACCA |
| 66 | >hsa-miR-378b MIMAT0014999 | TGTCGGACTGGACTTGGAG | ACUGGACUUGGAGGCAGAA | GTGCAGGGTCCGAGGTCAGAGCCACCTGGGCAATTTTTTTTTTTCTGCCT |
| 66 | >hsa-miR-382-3p MIMAT0022697 | GTCGGAATCATTCACGGACA | AAUCAUUCACGGACAACACUU | GTGCAGGGTCCGAGGTCAGAGCCACCTGGGCAATTTTTTTTTTTAAGTGT |
| 66 | >hsa-miR-92a-3p MIMAT0000092 | CGGTATTGCACTTGTCCCG | UAUUGCACUUGUCCCGGCCUGU | GTGCAGGGTCCGAGGTCAGAGCCACCTGGGCAATTTTTTTTTTTACAGGC |
| 66 | >hsa-miR-519b-5p MIMAT0005454 | GGCTCTAGAGGGAAGCGC | CUCUAGAGGGAAGCGCUUUCUG | GTGCAGGGTCCGAGGTCAGAGCCACCTGGGCAATTTTTTTTTTTCAGAAA |
| 66 | >hsa-miR-221-3p MIMAT0000278 | TGGAGCTACATTGTCTGCTG | AGCUACAUUGUCUGCUGGGUUUC | GTGCAGGGTCCGAGGTCAGAGCCACCTGGGCAATTTTTTTTTTTGAAACC |
| 66 | >hsa-miR-378f MIMAT0018932 | TTCGGACTGGACTTGGAGC | ACUGGACUUGGAGCCAGAAG | GTGCAGGGTCCGAGGTCAGAGCCACCTGGGCAATTTTTTTTTTTCTTCTG |
| 67 | >hsa-miR-369-5p MIMAT0001621 | TCGGAGATCGACCGTGTTAT | AGAUCGACCGUGUUAUAUUCGC | GTGCAGGGTCCGAGGTCAGAGCCACCTGGGCAATTTTTTTTTTTGCGAAT |
| 67 | >hsa-miR-548as-5p MIMAT0022267 | TTCGGAAAAGTAATTGCGGGT | AAAAGUAAUUGCGGGUUUUGCC | GTGCAGGGTCCGAGGTCAGAGCCACCTGGGCAATTTTTTTTTTTGGCAAA |
| 67 | >hsa-miR-329-3p MIMAT0001629 | TCGGAACACACCTGGTTAAC | AACACACCUGGUUAACCUCUUU | GTGCAGGGTCCGAGGTCAGAGCCACCTGGGCAATTTTTTTTTTTAAAGAG |
| 67 | >hsa-miR-522-5p MIMAT0005451 | GGCTCTAGAGGGAAGCGC | CUCUAGAGGGAAGCGCUUUCUG | GTGCAGGGTCCGAGGTCAGAGCCACCTGGGCAATTTTTTTTTTTCAGAAA |
| 67 | >hsa-miR-3680-3p MIMAT0018107 | GGTTTTGCATGACCCTGGG | UUUUGCAUGACCCUGGGAGUAGG | GTGCAGGGTCCGAGGTCAGAGCCACCTGGGCAATTTTTTTTTTTCCTACT |
| 67 | >hsa-miR-34b-5p MIMAT0000685 | TGGTAGGCAGTGTCATTAGC | UAGGCAGUGUCAUUAGCUGAUUG | GTGCAGGGTCCGAGGTCAGAGCCACCTGGGCAATTTTTTTTTTTCAATCA |
| 67 | >hsa-miR-3908 MIMAT0018182 | TCGGGAGCAATGTAGGTAGA | GAGCAAUGUAGGUAGACUGUUU | GTGCAGGGTCCGAGGTCAGAGCCACCTGGGCAATTTTTTTTTTTAAACAG |
| 68 | >hsa-miR-297 MIMAT0004450 | GTCGGATGTATGTGTGCATG | AUGUAUGUGUGCAUGUGCAUG | GTGCAGGGTCCGAGGTCAGAGCCACCTGGGCAATTTTTTTTTTTCATGCA |
| 68 | >hsa-miR-432-5p MIMAT0002814 | CGGTCTTGGAGTAGGTCATT | UCUUGGAGUAGGUCAUUGGGUGG | GTGCAGGGTCCGAGGTCAGAGCCACCTGGGCAATTTTTTTTTTTCCACCC |
| 68 | >hsa-miR-371a-5p MIMAT0004687 | GTCGGACTCAAACTGTGGG | ACUCAAACUGUGGGGGCACU | GTGCAGGGTCCGAGGTCAGAGCCACCTGGGCAATTTTTTTTTTTAGTGCC |
| 68 | >hsa-miR-3672 MIMAT0018095 | TTCGGATGAGACTCATGTAAAA | AUGAGACUCAUGUAAAACAUCUU | GTGCAGGGTCCGAGGTCAGAGCCACCTGGGCAATTTTTTTTTTTAAGATG |
| 68 | >hsa-miR-3667-3p MIMAT0018090 | TGGACCTTCCTCTCCATGG | ACCUUCCUCUCCAUGGGUCUUU | GTGCAGGGTCCGAGGTCAGAGCCACCTGGGCAATTTTTTTTTTTAAAGAC |
| 68 | >hsa-miR-181c-5p MIMAT0000258 | TCGGAACATTCAACCTGTCG | AACAUUCAACCUGUCGGUGAGU | GTGCAGGGTCCGAGGTCAGAGCCACCTGGGCAATTTTTTTTTTTACTCAC |
| 68 | >hsa-miR-3176 MIMAT0015053 | TTCGGACTGGCCTGGGAC | ACUGGCCUGGGACUACCGG | GTGCAGGGTCCGAGGTCAGAGCCACCTGGGCAATTTTTTTTTTTCCGGTA |
| 69 | >hsa-miR-635 MIMAT0003305 | TGGACTTGGGCACTGAAACA | ACUUGGGCACUGAAACAAUGUCC | GTGCAGGGTCCGAGGTCAGAGCCACCTGGGCAATTTTTTTTTTTGGACAT |
| 69 | >hsa-miR-548h-5p MIMAT0005928 | TTCGGAAAAGTAATCGCGGTT | AAAAGUAAUCGCGGUUUUUGUC | GTGCAGGGTCCGAGGTCAGAGCCACCTGGGCAATTTTTTTTTTTGACAAA |
| 69 | >hsa-miR-9-5p MIMAT0000441 | TCGGTCTTTGGTTATCTAGCT | UCUUUGGUUAUCUAGCUGUAUGA | GTGCAGGGTCCGAGGTCAGAGCCACCTGGGCAATTTTTTTTTTTCATACA |
| 69 | >hsa-miR-548c-3p MIMAT0003285 | TGTCGGCAAAAATCTCAATTAC | CAAAAAUCUCAAUUACUUUUGC | GTGCAGGGTCCGAGGTCAGAGCCACCTGGGCAATTTTTTTTTTTGCAAAA |
| 69 | >hsa-miR-335-5p MIMAT0000765 | TCGGTCAAGAGCAATAACGAA | UCAAGAGCAAUAACGAAAAAUGU | GTGCAGGGTCCGAGGTCAGAGCCACCTGGGCAATTTTTTTTTTTACATTT |
| 69 | >hsa-miR-1178 MIMAT0005823 | GTCGGTTGCTCACTGTTCTT | UUGCUCACUGUUCUUCCCUAG | GTGCAGGGTCCGAGGTCAGAGCCACCTGGGCAATTTTTTTTTTTCTAGGG |
| 69 | >hsa-miR-4454 MIMAT0018976 | TCGGGGATCCGAGTCACG | GGAUCCGAGUCACGGCACCA | GTGCAGGGTCCGAGGTCAGAGCCACCTGGGCAATTTTTTTTTTTGGTGCC |
| 70 | >hsa-miR-520a-3p MIMAT0002834 | TCGGAAAGTGCTTCCCTTTG | AAAGUGCUUCCCUUUGGACUGU | GTGCAGGGTCCGAGGTCAGAGCCACCTGGGCAATTTTTTTTTTTACAGTC |
| 70 | >hsa-miR-122-5p MIMAT0000421 | CGGTGGAGTGTGACAATGG | UGGAGUGUGACAAUGGUGUUUG | GTGCAGGGTCCGAGGTCAGAGCCACCTGGGCAATTTTTTTTTTTCAAACA |
| 70 | >hsa-miR-208a-3p MIMAT0000241 | GTCGGATAAGACGAGCAAAAA | AUAAGACGAGCAAAAAGCUUGU | GTGCAGGGTCCGAGGTCAGAGCCACCTGGGCAATTTTTTTTTTTACAAGC |
|  |  |  |  |  |
|  | >cel-miR-54-5p MIMAT0020773 | TCGGAGGATATGAGACGACG | AGGAUAUGAGACGACGAGAACA | GTGCAGGGTCCGAGGTCAGAGCCACCTGGGCAATTTTTTTTTTTGTTCTC |
|  | Universal reverse primer |  | CAGTGCAGGGTCCGAGGT |  |
|  | Universal Taqman probe |  | 56-FAM/CAGAGCCAC/ZEN/CTGGGCAATTT/3IABkFQ |  |
